# Perspectives on ENCODE

**DOI:** 10.1038/s41586-020-2449-8

**Published:** 2020-07-29

**Authors:** The ENCODE Project Consortium, Federico Abascal, Reyes Acosta, Nicholas J. Addleman, Jessika Adrian, Veena Afzal, Bronwen Aken, Jennifer A. Akiyama, Omar Al Jammal, Henry Amrhein, Stacie M. Anderson, Gregory R. Andrews, Igor Antoshechkin, Kristin G. Ardlie, Joel Armstrong, Matthew Astley, Budhaditya Banerjee, Amira A. Barkal, If H. A. Barnes, Iros Barozzi, Daniel Barrell, Gemma Barson, Daniel Bates, Ulugbek K. Baymuradov, Cassandra Bazile, Michael A. Beer, Samantha Beik, M. A. Bender, Ruth Bennett, Louis Philip Benoit Bouvrette, Bradley E. Bernstein, Andrew Berry, Anand Bhaskar, Alexandra Bignell, Steven M. Blue, David M. Bodine, Carles Boix, Nathan Boley, Tyler Borrman, Beatrice Borsari, Alan P. Boyle, Laurel A. Brandsmeier, Alessandra Breschi, Emery H. Bresnick, Jason A. Brooks, Michael Buckley, Christopher B. Burge, Rachel Byron, Eileen Cahill, Lingling Cai, Lulu Cao, Mark Carty, Rosa G. Castanon, Andres Castillo, Hassan Chaib, Esther T. Chan, Daniel R. Chee, Sora Chee, Hao Chen, Huaming Chen, Jia-Yu Chen, Songjie Chen, J. Michael Cherry, Surya B. Chhetri, Jyoti S. Choudhary, Jacqueline Chrast, Dongjun Chung, Declan Clarke, Neal A. L. Cody, Candice J. Coppola, Julie Coursen, Anthony M. D’Ippolito, Stephen Dalton, Cassidy Danyko, Claire Davidson, Jose Davila-Velderrain, Carrie A. Davis, Job Dekker, Alden Deran, Gilberto DeSalvo, Gloria Despacio-Reyes, Colin N. Dewey, Diane E. Dickel, Morgan Diegel, Mark Diekhans, Vishnu Dileep, Bo Ding, Sarah Djebali, Alexander Dobin, Daniel Dominguez, Sarah Donaldson, Jorg Drenkow, Timothy R. Dreszer, Yotam Drier, Michael O. Duff, Douglass Dunn, Catharine Eastman, Joseph R. Ecker, Matthew D. Edwards, Nicole El-Ali, Shaimae I. Elhajjajy, Keri Elkins, Andrew Emili, Charles B. Epstein, Rachel C. Evans, Iakes Ezkurdia, Kaili Fan, Peggy J. Farnham, Nina Farrell, Elise A. Feingold, Anne-Maud Ferreira, Katherine Fisher-Aylor, Stephen Fitzgerald, Paul Flicek, Chuan Sheng Foo, Kevin Fortier, Adam Frankish, Peter Freese, Shaliu Fu, Xiang-Dong Fu, Yu Fu, Yoko Fukuda-Yuzawa, Mariateresa Fulciniti, Alister P. W. Funnell, Idan Gabdank, Timur Galeev, Mingshi Gao, Carlos Garcia Giron, Tyler H. Garvin, Chelsea Anne Gelboin-Burkhart, Grigorios Georgolopoulos, Mark B. Gerstein, Belinda M. Giardine, David K. Gifford, David M. Gilbert, Daniel A. Gilchrist, Shawn Gillespie, Thomas R. Gingeras, Peng Gong, Alvaro Gonzalez, Jose M. Gonzalez, Peter Good, Alon Goren, David U. Gorkin, Brenton R. Graveley, Michael Gray, Jack F. Greenblatt, Ed Griffiths, Mark T. Groudine, Fabian Grubert, Mengting Gu, Roderic Guigó, Hongbo Guo, Yu Guo, Yuchun Guo, Gamze Gursoy, Maria Gutierrez-Arcelus, Jessica Halow, Ross C. Hardison, Matthew Hardy, Manoj Hariharan, Arif Harmanci, Anne Harrington, Jennifer L. Harrow, Tatsunori B. Hashimoto, Richard D. Hasz, Meital Hatan, Eric Haugen, James E. Hayes, Peng He, Yupeng He, Nastaran Heidari, David Hendrickson, Elisabeth F. Heuston, Jason A. Hilton, Benjamin C. Hitz, Abigail Hochman, Cory Holgren, Lei Hou, Shuyu Hou, Yun-Hua E. Hsiao, Shanna Hsu, Hui Huang, Tim J. Hubbard, Jack Huey, Timothy R. Hughes, Toby Hunt, Sean Ibarrientos, Robbyn Issner, Mineo Iwata, Osagie Izuogu, Tommi Jaakkola, Nader Jameel, Camden Jansen, Lixia Jiang, Peng Jiang, Audra Johnson, Rory Johnson, Irwin Jungreis, Madhura Kadaba, Maya Kasowski, Mary Kasparian, Momoe Kato, Rajinder Kaul, Trupti Kawli, Michael Kay, Judith C. Keen, Sunduz Keles, Cheryl A. Keller, David Kelley, Manolis Kellis, Pouya Kheradpour, Daniel Sunwook Kim, Anthony Kirilusha, Robert J. Klein, Birgit Knoechel, Samantha Kuan, Michael J. Kulik, Sushant Kumar, Anshul Kundaje, Tanya Kutyavin, Julien Lagarde, Bryan R. Lajoie, Nicole J. Lambert, John Lazar, Ah Young Lee, Donghoon Lee, Elizabeth Lee, Jin Wook Lee, Kristen Lee, Christina S. Leslie, Shawn Levy, Bin Li, Hairi Li, Nan Li, Xiangrui Li, Yang I. Li, Ying Li, Yining Li, Yue Li, Jin Lian, Maxwell W. Libbrecht, Shin Lin, Yiing Lin, Dianbo Liu, Jason Liu, Peng Liu, Tingting Liu, X. Shirley Liu, Yan Liu, Yaping Liu, Maria Long, Shaoke Lou, Jane Loveland, Aiping Lu, Yuheng Lu, Eric Lécuyer, Lijia Ma, Mark Mackiewicz, Brandon J. Mannion, Michael Mannstadt, Deepa Manthravadi, Georgi K. Marinov, Fergal J. Martin, Eugenio Mattei, Kenneth McCue, Megan McEown, Graham McVicker, Sarah K. Meadows, Alex Meissner, Eric M. Mendenhall, Christopher L. Messer, Wouter Meuleman, Clifford Meyer, Steve Miller, Matthew G. Milton, Tejaswini Mishra, Dianna E. Moore, Helen M. Moore, Jill E. Moore, Samuel H. Moore, Jennifer Moran, Ali Mortazavi, Jonathan M. Mudge, Nikhil Munshi, Rabi Murad, Richard M. Myers, Vivek Nandakumar, Preetha Nandi, Anil M. Narasimha, Aditi K. Narayanan, Hannah Naughton, Fabio C. P. Navarro, Patrick Navas, Jurijs Nazarovs, Jemma Nelson, Shane Neph, Fidencio Jun Neri, Joseph R. Nery, Amy R. Nesmith, J. Scott Newberry, Kimberly M. Newberry, Vu Ngo, Rosy Nguyen, Thai B. Nguyen, Tung Nguyen, Andrew Nishida, William S. Noble, Catherine S. Novak, Eva Maria Novoa, Briana Nuñez, Charles W. O’Donnell, Sara Olson, Kathrina C. Onate, Ericka Otterman, Hakan Ozadam, Michael Pagan, Tsultrim Palden, Xinghua Pan, Yongjin Park, E. Christopher Partridge, Benedict Paten, Florencia Pauli-Behn, Michael J. Pazin, Baikang Pei, Len A. Pennacchio, Alexander R. Perez, Emily H. Perry, Dmitri D. Pervouchine, Nishigandha N. Phalke, Quan Pham, Doug H. Phanstiel, Ingrid Plajzer-Frick, Gabriel A. Pratt, Henry E. Pratt, Sebastian Preissl, Jonathan K. Pritchard, Yuri Pritykin, Michael J. Purcaro, Qian Qin, Giovanni Quinones-Valdez, Ines Rabano, Ernest Radovani, Anil Raj, Nisha Rajagopal, Oren Ram, Lucia Ramirez, Ricardo N. Ramirez, Dylan Rausch, Soumya Raychaudhuri, Joseph Raymond, Rozita Razavi, Timothy E. Reddy, Thomas M. Reimonn, Bing Ren, Alexandre Reymond, Alex Reynolds, Suhn K. Rhie, John Rinn, Miguel Rivera, Juan Carlos Rivera-Mulia, Brian Roberts, Jose Manuel Rodriguez, Joel Rozowsky, Russell Ryan, Eric Rynes, Denis N. Salins, Richard Sandstrom, Takayo Sasaki, Shashank Sathe, Daniel Savic, Alexandra Scavelli, Jonathan Scheiman, Christoph Schlaffner, Jeffery A. Schloss, Frank W. Schmitges, Lei Hoon See, Anurag Sethi, Manu Setty, Anthony Shafer, Shuo Shan, Eilon Sharon, Quan Shen, Yin Shen, Richard I. Sherwood, Minyi Shi, Sunyoung Shin, Noam Shoresh, Kyle Siebenthall, Cristina Sisu, Teri Slifer, Cricket A. Sloan, Anna Smith, Valentina Snetkova, Michael P. Snyder, Damek V. Spacek, Sharanya Srinivasan, Rohith Srivas, George Stamatoyannopoulos, John A. Stamatoyannopoulos, Rebecca Stanton, Dave Steffan, Sandra Stehling-Sun, J. Seth Strattan, Amanda Su, Balaji Sundararaman, Marie-Marthe Suner, Tahin Syed, Matt Szynkarek, Forrest Y. Tanaka, Danielle Tenen, Mingxiang Teng, Jeffrey A. Thomas, Dave Toffey, Michael L. Tress, Diane E. Trout, Gosia Trynka, Junko Tsuji, Sean A. Upchurch, Oana Ursu, Barbara Uszczynska-Ratajczak, Mia C. Uziel, Alfonso Valencia, Benjamin Van Biber, Arjan G. van der Velde, Eric L. Van Nostrand, Yekaterina Vaydylevich, Jesus Vazquez, Alec Victorsen, Jost Vielmetter, Jeff Vierstra, Axel Visel, Anna Vlasova, Christopher M. Vockley, Simona Volpi, Shinny Vong, Hao Wang, Mengchi Wang, Qin Wang, Ruth Wang, Tao Wang, Wei Wang, Xiaofeng Wang, Yanli Wang, Nathaniel K. Watson, Xintao Wei, Zhijie Wei, Hendrik Weisser, Sherman M. Weissman, Rene Welch, Robert E. Welikson, Zhiping Weng, Harm-Jan Westra, John W. Whitaker, Collin White, Kevin P. White, Andre Wildberg, Brian A. Williams, David Wine, Heather N. Witt, Barbara Wold, Maxim Wolf, James Wright, Rui Xiao, Xinshu Xiao, Jie Xu, Jinrui Xu, Koon-Kiu Yan, Yongqi Yan, Hongbo Yang, Xinqiong Yang, Yi-Wen Yang, Galip Gürkan Yardımcı, Brian A. Yee, Gene W. Yeo, Taylor Young, Tianxiong Yu, Feng Yue, Chris Zaleski, Chongzhi Zang, Haoyang Zeng, Weihua Zeng, Daniel R. Zerbino, Jie Zhai, Lijun Zhan, Ye Zhan, Bo Zhang, Jialing Zhang, Jing Zhang, Kai Zhang, Lijun Zhang, Peng Zhang, Qi Zhang, Xiao-Ou Zhang, Yanxiao Zhang, Zhizhuo Zhang, Yuan Zhao, Ye Zheng, Guoqing Zhong, Xiao-Qiao Zhou, Yun Zhu, Jared Zimmerman, Michael P. Snyder, Thomas R. Gingeras, Jill E. Moore, Zhiping Weng, Mark B. Gerstein, Bing Ren, Ross C. Hardison, John A. Stamatoyannopoulos, Brenton R. Graveley, Elise A. Feingold, Michael J. Pazin, Michael Pagan, Daniel A. Gilchrist, Benjamin C. Hitz, J. Michael Cherry, Bradley E. Bernstein, Eric M. Mendenhall, Daniel R. Zerbino, Adam Frankish, Paul Flicek, Richard M. Myers

**Affiliations:** 1 Department of Genetics, School of Medicine, Stanford University, Palo Alto, CA USA; 2 Cardiovascular Institute, Stanford School of Medicine, Stanford, CA USA; 3 Functional Genomics, Cold Spring Harbor Laboratory, Cold Spring Harbor, NY USA; 4 University of Massachusetts Medical School, Program in Bioinformatics and Integrative Biology, Worcester, MA USA; 5 Department of Thoracic Surgery, Clinical Translational Research Center, Shanghai Pulmonary Hospital, The School of Life Sciences and Technology, Tongji University, Shanghai, China; 6 Bioinformatics Program, Boston University, Boston, MA USA; 7 Yale University, New Haven, CT USA; 8 Ludwig Institute for Cancer Research, University of California, San Diego, La Jolla, CA USA; 9 Center for Epigenomics, University of California, San Diego, La Jolla, CA USA; 10 Department of Biochemistry and Molecular Biology, The Pennsylvania State University, University Park, PA USA; 11 Altius Institute for Biomedical Sciences, Seattle, WA USA; 12 Department of Genome Sciences, University of Washington, Seattle, WA USA; 13 Department of Medicine, University of Washington, Seattle, WA USA; 14 Department of Genetics and Genome Sciences, Institute for Systems Genomics, UConn Health, Farmington, CT USA; 15 National Human Genome Research Institute, National Institutes of Health, Bethesda, MD USA; 16 Broad Institute and Department of Pathology, Massachusetts General Hospital and Harvard Medical School, Boston, MA USA; 17 Biological Sciences, University of Alabama in Huntsville, Huntsville, AL USA; 18 HudsonAlpha Institute for Biotechnology, Huntsville, AL USA; 19 European Molecular Biology Laboratory, European Bioinformatics Institute, Wellcome Genome Campus, Cambridge, UK; 20 The Broad Institute of Harvard and MIT, Cambridge, MA USA; 21 MGH, Boston, MA USA; 22 Dana-Farber Cancer Institute, Boston, MA USA; 23 Harvard Medical School, Boston, MA USA; 24 Boston Children’s Hospital, Boston, MA USA; 25 Harvard University, Cambridge, MA USA; 26 Computer Science and Artificial Intelligence Laboratory, Massachusetts Institute of Technology, Cambridge, MA USA; 27 Max Planck Institute for Molecular Genetics, Department of Genome Regulation, Berlin, Germany; 28 University of Colorado Boulder, Boulder, CO USA; 29 Bioinformatics and Genomics Program, Centre for Genomic Regulation (CRG), The Barcelona Institute of Science and Technology and Universitat Pompeu Fabra, Barcelona, Spain; 30 IRSD, Université de Toulouse, INSERM, INRA, ENVT, UPS, U1220, CHU Purpan, CS60039 Toulouse, France; 31 Skolkovo Institute for Science and Technology, Moscow, Russia; 32 Functional Genomics, Cold Spring Harbor Laboratory, Woodbury, NY USA; 33 Department of Clinical Research, University of Bern, Bern, Switzerland; 34 International Institute of Molecular and Cell Biology, Warsaw, Poland; 35 Department of Biology, Massachusetts Institute of Technology, Cambridge, MA USA; 36 Department of Cellular and Molecular Medicine, Institute for Genomic Medicine, Stem Cell Program, Sanford Consortium for Regenerative Medicine, University of California, San Diego, La Jolla, CA USA; 37 Département de Biochimie et Médecine Moléculaire, Université de Montréal, Montréal, Quebec, Canada; 38 Division of Experimental Medicine, McGill University, Quebec, Canada; 39 Institut de Recherches Cliniques de Montréal (IRCM), Montréal, Quebec Canada; 40 Program in Computational and Systems Biology, Massachusetts Institute of Technology, Cambridge, MA USA; 41 Department of Cellular and Molecular Medicine, Institute of Genomic Medicine, University of California, San Diego, San Diego, CA USA; 42 Department of Pharmaceutical Sciences, St. Jude Children’s Research Hospital, Memphis, TN USA; 43 Division of Biology, California Institute of Technology, Pasadena, CA USA; 44 National Human Genome Research Institute, National Institutes of Health, Bethesda, MD USA; 45 Department of Biostatistics and Bioinformatics, Duke University, Durham, NC USA; 46 Center for Genomic and Computational Biology, Duke University, Durham, NC USA; 47 Biological Sciences, University of California, Irvine, Irvine, CA USA; 48 Salk Institute for Biological Studies, La Jolla, CA USA; 49 Environmental Genomics and Systems Biology Division, Lawrence Berkeley National Laboratory, Berkeley, CA USA; 50 Department of Chemistry and Biochemistry, Department of Cellular and Molecular Medicine, UC San Diego, La Jolla, CA USA; 51 Institute for Human Genetics, Department of Neurology, University of California, San Francisco, San Francisco, CA USA; 52 Department of Biochemistry and Molecular Genetics, Northwestern University Feinberg School of Medicine, Chicago, IL USA; 53 Penn State Health Milton S. Hershey Medical Center, Hershey, PA USA; 54 US Department of Energy Joint Genome Institute, Lawrence Berkeley National Laboratory, Berkeley, CA USA; 55 School of Natural Sciences, University of California, Merced, Merced, CA USA; 56 Comparative Biochemistry Program, University of California, Berkeley, CA USA; 57 Howard Hughes Medical Institute, The Salk Institute for Biological Studies, La Jolla, CA USA; 58 Department of Computational Medicine and Bioinformatics, University of Michigan, Ann Arbor, MI USA; 59 Department of Human Genetics, University of Michigan, Ann Arbor, MI USA; 60 Department of Molecular and Cellular Physiology, School of Medicine, Stanford University, Palo Alto, CA USA; 61 Terrence Donnelly Centre for Cellular and Biomolecular Research, University of Toronto, Toronto, Ontario Canada; 62 Department of Genetics, School of Medicine, Yale University, New Haven, CT USA; 63 Department of Biochemistry and Molecular Medicine, Norris Comprehensive Cancer Center, Keck School of Medicine, University of Southern California, Los Angeles, CA USA; 64 Department of Radiation Oncology, School of Medicine, Stanford University, Palo Alto, CA USA; 65 Department of Human Genetics, Institute for Genomics and Systems Biology, The University of Chicago, Chicago, IL USA; 66 Division of General Surgery, Section of Transplant Surgery, School of Medicine, Washington University, St. Louis, MO USA; 67 Department of Biochemistry and Molecular Biology, School of Basic Medical Sciences, Southern Medical University, Guangzhou, China; 68 Guangdong Provincial Key Laboratory of Single Cell Technology and Application, Guangzhou, China; 69 Department of Cell Biology & Physiology, University of North Carolina at Chapel Hill, Chapel Hill, NC USA; 70 Thurston Arthritis Research Center, University of North Carolina at Chapel Hill, Chapel Hill, NC USA; 71 Department of Molecular Genetics, University of Toronto, Toronto, Ontario Canada; 72 School of Medicine, Jiangsu University, Zhenjiang, China; 73 Department of Molecular Genetics, Donnelly Centre, University of Toronto, Toronto, Ontario Canada; 74 Tempus Labs, Chicago, IL USA; 75 Department of Medicine, University of Washington, Seattle, WA USA; 76 Fred Hutchinson Cancer Research Center, Seattle, WA USA; 77 HHMI and Program in Systems Biology, University of Massachusetts Medical School, Albert Sherman Center, Worcester, MA USA; 78 University of Massachusetts Amherst, Amherst, MA USA; 79 Institute for Infocomm Research, Singapore, Singapore; 80 Simon Fraser University, Burnaby, British Columbia Canada; 81 Center for Functional Cancer Epigenetics, Dana-Farber Cancer Institute, Boston, MA USA; 82 Department of Data Sciences, Dana-Farber Cancer Institute and Harvard T.H. Chan School of Public Health, Boston, MA USA; 83 Center for Public Health Genomics, University of Virginia, Charlottesville, VA USA; 84 Molecular Pathology Unit & Cancer Center, Boston, MA USA; 85 Department of Biostatistics and Bioinformatics, Moffitt Cancer Center, Tampa, FL USA; 86 Institute for Stem Cell Biology and Regenerative Medicine, Stanford University, Stanford, CA USA; 87 Department of Medicine, Brigham and Women’s Hospital and Harvard Medical School, Boston, MA USA; 88 Department of Statistics, Medical Sciences Center, Madison, WI USA; 89 Department of Biostatistics and Medical Informatics, Madison, WI USA; 90 Department of Mathematical Sciences, University of Texas at Dallas, Richardson, TX USA; 91 Department of Statistics, University of Nebraska-Lincoln, Lincoln, NE USA; 92 Department of Biomedical Informatics, The Ohio State University, Columbus, OH USA; 93 Department of Cell and Regenerative Biology, UW-Madison Blood Research Program, Carbone Cancer Center, University of Wisconsin School of Medicine and Public Health, University of Wisconsin, Madison, WI USA; 94 Department of Genetics and Genomic Sciences, Icahn School of Medicine at Mount Sinai, New York, NY USA; 95 Wellcome Sanger Institute, Cambridge, UK; 96 Program in Computational Biology, Memorial Sloan Kettering Cancer Center, New York, NY USA; 97 610 Charles E. Young Drive S, Terasaki Life Sciences Building, Room 2000E, Los Angeles, CA USA; 98 McKusick-Nathans Institute of Genetic Medicine, Johns Hopkins University, Baltimore, MD USA; 99 Department of Biomedical Engineering, Johns Hopkins University, Baltimore, MD USA; 100 University of California, Santa Cruz, Santa Cruz, CA USA; 101 Center for Integrative Genomics, University of Lausanne, Lausanne, Switzerland; 102 Centro Nacional de Investigaciones Cardiovasculares (CNIC) and CIBER de Enfermedades Cardiovasculares (CIBERCV), Madrid, Spain; 103 Spanish National Cancer Research Centre (CNIO), Madrid, Spain; 104 Brunel University London, London, UK; 105 King’s College London, Guy’s Hospital, London, UK; 106 ELIXIR Hub, Wellcome Genome Campus, Cambridge, UK; 107 Institute of Cancer Research, London, UK; 108 Department of Biological Science, Florida State University, Tallahassee, FL USA; 109 Department of Biochemistry, Molecular Biology and Biophysics, University of Minnesota Medical School, Minneapolis, MN USA; 110 Center for Vaccines and Immunology University of Georgia, Athens, GA USA; 111 Center for Molecular Medicine and Department of Biochemistry and Molecular Biology, University of Georgia, Athens, GA USA; 112 Gift of Life Donor Program, Philadelphia, PA USA; 113 American Society for Radiation Oncology, Arlington, VA USA; 114 National Cancer Institute, National Institutes of Health, Bethesda, MD USA; 115 Leidos Biomedical, Inc, Frederick, MD USA; 116 National Disease Research Interchange (NDRI), Philadelphia, PA USA; 117 4407 Puller Drive, Kensington, MD USA

**Keywords:** Epigenomics, Genome, Epigenetics, Transcriptomics

## Abstract

The Encylopedia of DNA Elements (ENCODE) Project launched in 2003 with the long-term goal of developing a comprehensive map of functional elements in the human genome. These included genes, biochemical regions associated with gene regulation (for example, transcription factor binding sites, open chromatin, and histone marks) and transcript isoforms. The marks serve as sites for candidate *cis*-regulatory elements (cCREs) that may serve functional roles in regulating gene expression^1^. The project has been extended to model organisms, particularly the mouse. In the third phase of ENCODE, nearly a million and more than 300,000 cCRE annotations have been generated for human and mouse, respectively, and these have provided a valuable resource for the scientific community.

## Abstract (web summary)

The authors summarize the history of the ENCODE Project, the achievements of ENCODE 1 and ENCODE 2, and how the new data generated and analysed in ENCODE 3 complement the previous phases.

## Main

The ENCODE Project was launched in 2003, as the first nearly complete human genome sequence was reported^2^. At that time, our understanding of the human genome was limited. For example, although 5% of the genome was known to be under purifying selection in placental mammals^3,4^, our knowledge of specific elements, particularly with regards to non-protein coding genes and regulatory regions, was restricted to a few well-studied loci^2,5^.

ENCODE commenced as an ambitious effort to comprehensively annotate the elements in the human genome, such as genes, control elements, and transcript isoforms, and was later expanded to annotate the genomes of several model organisms. Mapping assays identified biochemical activities and thus candidate regulatory elements.

**Extended Data Fig. 1:**
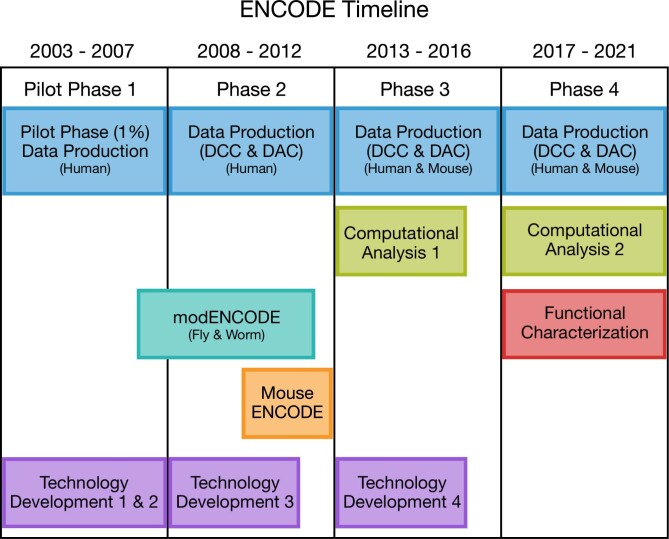
ENCODE timeline. Pilot phase: September 2003–September 2007; ENCODE 2: September 2007–September 2012; ENCODE 3: September 2012–January 2017; ENCODE 4: February 2017–present; modENCODE: April 2007–April 2012; mouse ENCODE: 2009–2012.

Analyses of the human genome in ENCODE proceeded in successive phases (Extended Data Fig. 1). Phase I (2003–2007) interrogated a specified 1% of the human genome in order to evaluate emerging technologies^6^. Half of this 1% was in regions of high interest, and the other half was chosen to sample the range of genomic features (such as G+C content and genes). Microarray-based assays were used to map transcribed regions, open chromatin, and regions associated with transcription factors and histone modification in a wide variety of cell lines, and these assays began to reveal the basic organizational features of the human genome and transcriptome. Phase II (2007–2012) introduced sequencing-based technologies (for example, chromatin immunoprecipitation with sequencing (ChIP–seq) and RNA sequencing (RNA-seq)) that interrogated the whole human genome and transcriptome^7^. General assays such as transcript, open-chromatin and histone modification mapping were used on a wide variety of cell lines, while more specific assays, such as mapping transcription factor binding regions, were performed extensively on a smaller number of cell lines to provide detailed annotations on, and to investigate the relationships of, many regulatory proteins across the genome. Transcriptome analysis of subcellular compartments (the nucleus, cytosol and subnuclear compartments) of these cells enabled the locations of transcripts to be analysed^7^.

## ENCODE phase III

**Fig. 1:**
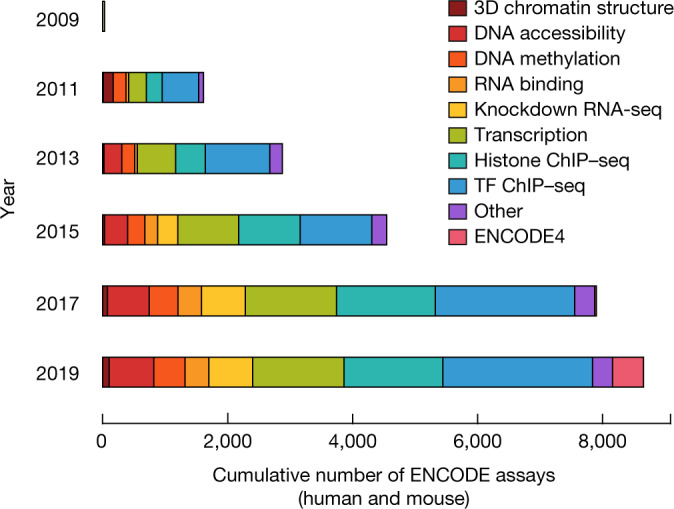
ENCODE assays by year. Accumulations of assays over the three phases of ENCODE. 3D chromatin structure includes ChIA-PET (62 experiments), Hi-C (31), and chromatin conformation capture carbon copy (5C, 13). Chromatin accessibility includes DNAase-seq (524), assay for transposase-accessible chromatin using sequencing (ATAC-seq, 129), transcription activator-like effector nuclease (TALEN)-modified DNAase-seq (40), formaldehyde-assisted isolation of regulator elements with sequencing (FAIRE-seq, 37) and micrococcal nuclease digestion with deep sequencing (MNase-seq, 2). DNA methylation includes DNAme arrays (259), WGBS (124), reduced-representation bisulfite sequencing (RRBS, 103), methylation-sensitive restriction enzyme sequencing (MRE-seq, 24) and methylated DNA immunoprecipitation coupled with next-generation sequencing (MeDIP-seq, 4). Histone modification includes ChIP–seq (1,605) on histone and modified histone targets. Knockdown transcription includes RNA-seq preceded by small interfering RNA (siRNA, 54), short hairpin RNA (shRNA, 531), clustered regularly interspaced short palindromic repeats (CRISPR, 50) or CRISPR interference (CRISPRi, 77). RNA binding includes enhanced cross-linking immunoprecipitation (eCLIP, 349), RNA bind-n-seq (158), RNA immunoprecipitation sequencing (RIP-seq, 158), RNA-binding protein immunoprecipitation-microarray profiling (RIP-chip, 32), individual nucleotide-resolution CLIP (iCLIP, 6) and Switchgear (2). Transcription includes RNA annotation and mapping of promoters for the analysis of gene expression (RAMPAGE, 155), cap analysis gene expression (CAGE, 78), RNA paired-end tag (RNA-PET, 31), microRNA-seq (114), microRNA counts (114), more classical RNA-seq (900) and RNA-microarray (170), including 112 experiments at single-cell resolution. Transcription factor (TF) binding is ChIP–seq on non-histone targets (2,443). Other assays include genotyping array (123), nascent DNA replication strand sequencing (Repli-seq, 104), replication strand arrays (Repli-chip, 63), tandem mass spectrometry (MS/MS, 14), genotyping by high-throughput sequencing (genotyping HTS, 12) and DNA-PET (6) can be looked at in detail at https://www.encodeproject.org.

**Fig. 2:**
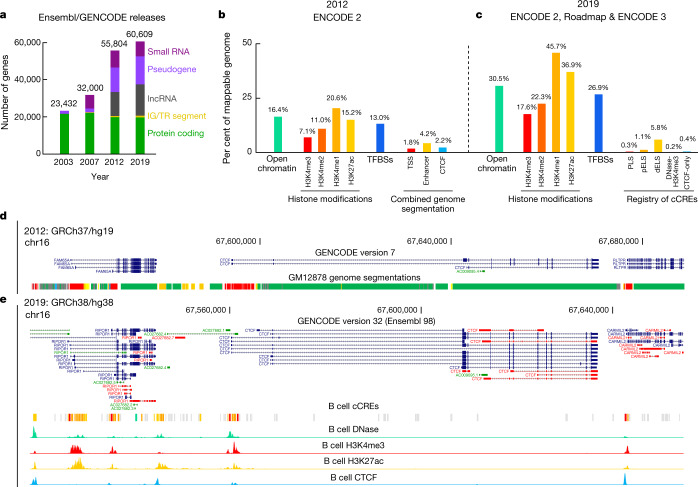
Progress in annotating the human genome. Link to high-resolution PDF file: https://www.dropbox.com/s/rjdrcqygz15p034/perspective.pdf?dl=0. **a**, Improvement of gene annotations in the past 15 years by GENCODE, an international gene annotation group that uses ENCODE data^42^. **b**, ENCODE annotations in 2012 with phase II data. Bars show the percentages of the mappable human genome (3.1 billion nucleotides; hg19) that were annotated as open chromatin by DNase-seq data, enriched in four types of active histone mark according to ChIP–seq data, and annotated as transcription factor binding sites (TFBSs) according to ChIP–seq data. Also shown are percentages of the genome assigned as transcription start sites (TSSs), enhancers and the insulator-binding protein (CTCF) by combining ChromHMM and Segway genome segmentations^7^. **c**, ENCODE annotations in 2019 with ENCODE 2, Roadmap, and ENCODE 3 data. The registry of cCREs developed during phase III defines 0.3%, 1.1%, 5.8%, 0.2% and 0.4% of the human genome as cCREs with promoter-like signatures (PLS), proximal enhancer-like signatures (pELS), distal enhancer-like signatures (dELS), with high DNase, high H3K4me3 and low H3K27ac signals (DNase-H3K4me3), and bound by CTCF, respectively. **d**, A UCSC genome browser view of GENCODE genes (V7) coloured by transcript annotation (blue for coding, green for noncoding, and red for problematic) and combined genome segmentation (TSSs in red, enhancers in orange, weak enhancers in yellow, transcription in green, repressed in grey) at the *CTCF* locus on the hg19 human genome. **e**, The UCSC genome browser view of GENCODE genes (V28, coloured as in **d**) and cCREs at the *CTCF* locus on the hg38 human genome^8^. Promoter-like, enhancer-like, and CTCF-only cCREs annotated in B cells are in red, yellow, and blue, respectively. The last four tracks show the DNase, H3K4me3, H3K27ac, and CTCF signals in B cells.

ENCODE 3 (2012–2017) expanded production and added new types of assays^8^ (Fig. 1, Extended Data Fig. 1), which revealed landscapes of RNA binding and the 3D organization of chromatin via methods such as chromatin interaction analysis by paired-end tagging (ChIA-PET) and Hi-C chromosome conformation capture. Phases 2 and 3 delivered 9,239 experiments (7,495 in human and 1,744 in mouse) in more than 500 cell types and tissues, including mapping of transcribed regions and transcript isoforms, regions of transcripts recognized by RNA-binding proteins, transcription factor binding regions, and regions that harbour specific histone modifications, open chromatin, and 3D chromatin interactions. The results of all of these experiments are available at the ENCODE portal (http://www.encodeproject.org). These efforts, combined with those of related projects and many other laboratories, have produced a greatly enhanced view of the human genome (Fig. 2), identifying 20,225 protein-coding and 37,595 noncoding genes (Fig. 2a), 2,157,387 open chromatin regions, 750,392 regions with modified histones (mono-, di- or tri-methylation of histone H3 at lysine 4 (H3K4me1, H3K4me2 or H3K4me3), or acetylation of histone 3 at lysine 27 (H3K27ac)), 1,224,154 regions bound by transcription factors and chromatin-associated proteins (Fig. 2c), 845,000 RNA subregions occupied by RNA-binding proteins, and more than 130,000 long-range interactions between chromatin loci. These annotations have greatly enhanced our view of the human genome from its original annotation in 2003 to a much richer and higher-resolution view (for example, Fig. 2d, e). Indeed, although the number of human protein-coding genes known has changed only modestly, the number of transcript isoforms, long noncoding RNAs (lncRNAs), and potential regulatory regions identified has increased greatly since the project began (Fig. 2a–c). An important part of ENCODE 3 is that the regulatory mapping efforts have now been integrated and synthesized into the first version of an encyclopedia, highlighting a registry of 0.9 million cCREs in human and 0.3 million cCREs in mouse. Details can be found in the accompanying ENCODE paper^8^ and companion papers in this issue and other journals^9–14^.

## Technology, quality control and standards

Reaching the present annotation required a substantial expansion of technology development, from ENCODE groups and others, as well as the establishment of standards to ensure that the data are reproducible and of high quality. Most ENCODE 2 assays used sequence-based readouts (for example, RNA-seq^15,16^ and ChIP–seq^17,18^) rather than the array-based methods^19,20^ used in the pilot phase, and in ENCODE 3, methods such as global mapping of 3D interactions^13^ and RNA-binding regions^14^ were added. Throughout the project, computational and visualization approaches were developed for mapping reads and integrating different data types (Supplementary Note 1).

A key feature of ENCODE is the application of data standards, including the use of independent replicates (separate experiments on two or more biological samples^5,21^), except when precluded by the limited availability of materials (for example, postmortem human tissues). Of the 8,699 ENCODE 2 and ENCODE 3 experiments, 6,101 have independent replicates. Of equal importance was the use of well-characterized reagents, such as antibodies for mapping sites of transcription factor binding, chromatin modifications and protein–RNA interactions^22^. ENCODE developed protocols to test each antibody ‘lot’ to demonstrate their experimental suitability, captured extensive metadata, and implemented controlled vocabularies and ontologies. Standards for reagents, experimental data, and metadata are on the ENCODE website: https://www.encodeproject.org/data-standards/.

Many metrics, including sequencing depth, mapping characteristics, replicate concordance, library complexity, and signal-to-noise ratio, were used to monitor the quality of each data set, and quality thresholds were applied^21^. A minority of experiments that fell short of the standards (for example, insufficiently validated antibodies) are still reported, but are marked with a badge to indicate that an issue was found. This is a compromise for having some data versus none when an experiment did not meet ENCODE-defined thresholds.

An important component is uniform data processing. Data from the major ENCODE assays (ChIP–seq, DNase I hypersensitive sites sequencing (DNase-seq), RNA-seq, and whole-genome bisulfite sequencing (WGBS)) are uniformly processed and the processing pipelines are available for users to apply to their own data, by downloading the code from the GitHub (http://github.com/ENCODE-DCC) or by accessing the pipelines at the DNAnexus cloud provider. The standards and pipelines will continue to evolve as new technologies arise and are implemented.

The ENCODE Consortium is a good example of how large-scale group efforts can have a large impact on the scientific community, and many other national and international projects—including the NIH Roadmap Epigenomics Program, The Cancer Genome Atlas (TCGA), the International Human Epigenome Consortium (IHEC), BLUEPRINT, the Canadian Epigenetics, Environment and Health Research Consortium (CEEHRC), the Genotype and Tissue Expression Project (GTEx), PsychENCODE, Functional Annotation of Animal Genomes (FAANG), the Global Alliance for Genomics and Health (GA4GH), the 4D Nucleome Program (4DN), the Human Cell Atlas and the FANTOM consortium—have now formed (Supplementary Note 1). ENCODE has engaged with most of these consortia to share standards for data quality control, submission, and uniform processing and has helped to facilitate the use of common ontologies with some of these consortia. Data from the now-completed NIH Roadmap Epigenomics Program have been reprocessed and are available in the ENCODE database and are part of the Encyclopedia annotation. ENCODE continues to work with other consortia, individually and as part of the IHEC and GA4GH (for example, http://epishare-project.org) to increase data interoperability and the value of its resources.

## ENCODE as a resource

**Fig. 3:**
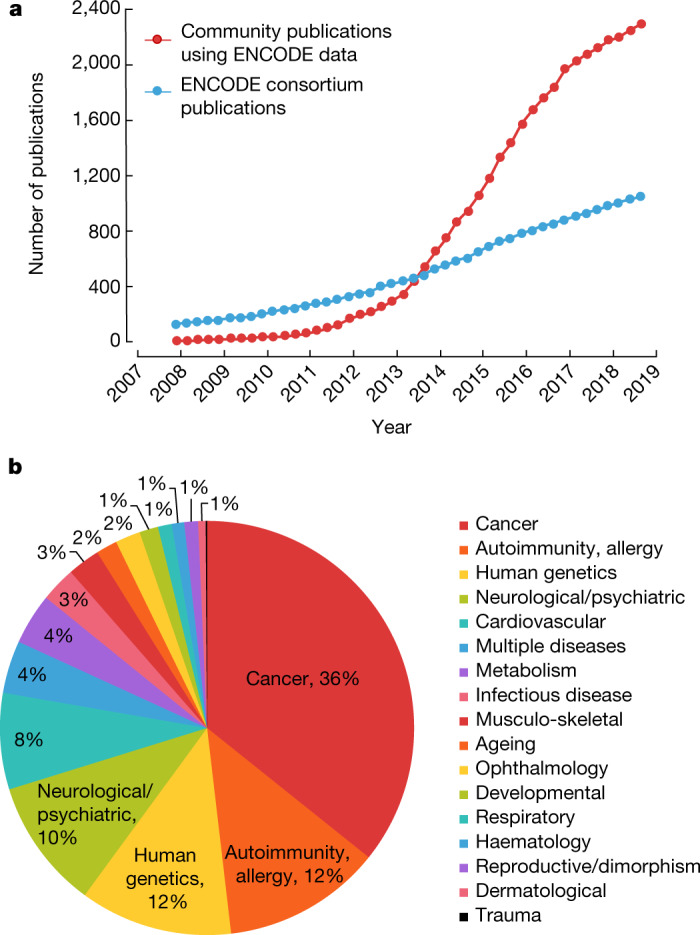
Publications using ENCODE data. The National Human Genome Research Institute (NHGRI) has identified a list of publications that used ENCODE data. This list is publicly shared to provide examples illustrating how the resource has been used (https://www.encodeproject.org/publications/). **a**, Publications over time. Community publications appear to use ENCODE data and do not report ENCODE grant support in PubMed; consortium publications report ENCODE grant support in PubMed. In brief, community publications are identified using two steps; first, candidates are identified through automated searches for citation of ENCODE accession numbers, ENCODE flagship papers, or resources such as HaploReg and RegulomeDB; second, candidates are manually evaluated to determine whether ENCODE data were actually used. Consortium papers are identified through automated searches of PubMed for publications that were supported at least in part by ENCODE awards, and are not further evaluated or annotated. **b**, Human disease example publications. The subset of community publications that were annotated as ‘human disease’ (other categories are basic biology, software tool, fly/worm data) were further manually categorized by disease aetiology.

The purpose of ENCODE is to provide valuable, accessible resources to the community. ENCODE data and derived features are available from a publicly accessible data portal (https://www.encodeproject.org), and consent was obtained from donors to make data freely available to the public. Raw and processed data are available directly from the cloud as an Amazon Public Data Set (https://registry.opendata.aws/encode-project/). The data are widely used by the scientific community—more than 2,000 publications from researchers outside of ENCODE have used ENCODE data to study diverse topics (Fig. 3). Because most disease-associated common variants are noncoding and show substantial enrichment in candidate cell-type-specific *cis* regulatory elements^23,24^, ENCODE-derived resources, both in isolation and in conjunction with data from other resources (for example, GTEx), can help to identify and interpret disease-associated noncoding variants (Fig. 3a). Users engage with the data in many ways, ranging from downloads of multiple data sets to detailed investigations of specific loci. Anyone navigating a major genome browser has access to thousands of biochemical, functional, and computational annotations to display at any genomic scale or to overlay on any sequence variant. Maps of epigenomic features relevant to gene regulation have been integrated to form a registry of discrete elements that are candidates for enhancers, promoters, or other regulatory elements. A specialized browser, SCREEN (http://screen.encodeproject.org), is an interface that can be used to identify and study these cCREs and associated ENCODE data and other annotations. This dynamic registry will be regularly updated as additional information is acquired.

## Mouse ENCODE and modENCODE

Model organism studies have produced essential insights into almost every aspect of biology, including genome organization and function. During ENCODE 2, mapping of mouse epigenomic and transcriptomic features was conducted in adult mouse tissues and cell lines through the Mouse ENCODE Project^25^, which identified 21,978 protein-coding regions, 32,168 noncoding genes, 1,192,301 open chromatin regions, 722,334 regions with modified histones H3K4me1, H3K4me2, H3K4me3, or H3K27ac, and 686,294 regions bound by transcription factors.

During ENCODE 2, a model organism ENCODE project (modENCODE^26,27^) was conducted to characterize the transcriptome, epigenome, and transcription factor binding sites in *Drosophila melanogaster* and *Caenorhabditis elegans* tissues, developmental stages and cell lines (Extended Data Fig. 1). These organisms provided the opportunity to develop detailed records of epigenomic features and transcriptome maps throughout development, which is difficult to accomplish in humans. Deep mapping of the spatial and temporal transcriptomes of these species has substantially enhanced the annotation of both genomes. Similarly, detailed mapping of the regulatory circuits that govern gene regulation in *Drosophila* and *C. elegans* has provided insights into general principles of genome organization and function. Mapping of transcription factor binding sites in *Drosophila* and *C. elegans* has continued after modENCODE ended in a project called model organism Encyclopedia of Regulatory Networks (modERN) and to date has characterized more than 262 transcription factors in *Drosophila* and 217 transcription factors in *C. elegans*^28^. Collectively, the modENCODE Project has provided new insights about how the genomes of multicellular organisms direct development and maintain homeostasis.

**Fig. 4:**
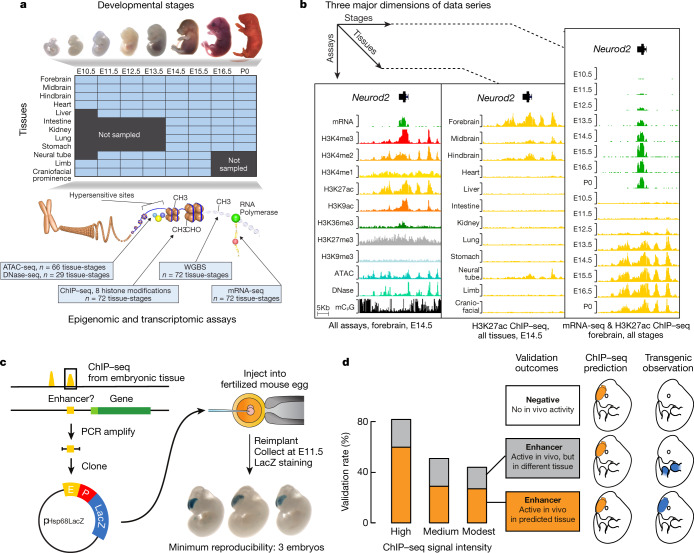
An overview of the mouse ENCODE Project in the current phase. **a**, Schematic representation of ENCODE 3 mouse developmental data series. The chromatin graphic is adapted from an image by Darryl Leja (NHGRI), Ian Dunham (EBI), and M.J.P. (NHGRI). The embryo image second from the right in was adapted from ref. ^43^, an Open Access article distributed under the terms of the Creative Commons Attribution License 2.0. **b**, Three major axes of the data series: assays, tissues, and developmental stages. The region shown is chr11:98,307,637–98,344,383, mm10. **c**, A schematic diagram of the transgenic assays used to validate and characterize the function of cCREs in E11.5 and E12.5 mouse embryos. The cCREs were selected on the basis of ChIP–seq data and cloned into a reporter vector that was then introduced into fertilized mouse eggs. The activities of the CRE were validated by tissue-specific expression patterns of the reporter gene. **d**, Results from recent transgenic assays^8,29^ to validate about 400 cCREs are summarized in a barchart, with the bars indicating the proportion of candidate CREs in each rank tier that showed reproducible reporter staining in the expected tissue (grey) or any tissue (pink).

In ENCODE phase III, experiments were carried out to characterize dynamic histone marks and accessibility, DNA methylomes, and transcriptomes in samples taken during eight mouse fetal developmental stages with up to twelve tissues per stage^28–30^ (Fig. 4). The resulting more than 1,500 datasets comprise, to our knowledge, the most comprehensive study of epigenomes and transcriptomes during the prenatal development of a mammal. Integrative analysis of these datasets has expanded our knowledge of the transcriptional regulatory networks that regulate mammalian development and underscored the role of gene regulatory mechanisms in human disease. At least 214,264 of the candidate enhancers identified in fetal mouse tissues are conserved in the human genome^8^. The human orthologues of these potential regulatory elements are significantly enriched for genetic variants that are associated with common illnesses in a tissue-restricted manner, providing information for investigations of the molecular basis of human disease^29,30^.

The mouse data from ENCODE 3 also include the results of more than 400 experiments using transgenic reporter mice designed to assess the function of cCREs in three embryonic tissues at two developmental stages. The results of this systematic study have helped to predict the in vivo activities of cCREs. For example, stronger enrichment for epigenetic signatures of enhancer activity correlated with higher rates of validation in the corresponding tissue^29,31^.

Finally, comparisons of epigenome and transcriptome maps across species have led to insights into the evolution of transcribed regions and regulatory information^25,32^. Combinatorial histone modification patterns at *cis*-regulatory elements and other genomic features are broadly conserved in metazoans. These chromatin states and transcript levels are highly correlated across tissues and developmental stages in all species examined. However, a notable fraction of specific *cis*-regulatory elements undergoes sequence and functional turnover during evolution, indicating that some regulatory components show substantial plasticity in their evolution while operating in a conserved regulatory network^33^.

## Current limitations: phase IV and beyond

It is now apparent that elements that govern transcription, chromatin organization, splicing, and other key aspects of genome control and function are densely encoded in the human genome; however, despite the discovery of many new elements, the annotation of elements that are highly selective for particular cell types or states is lagging behind. For example, very few examples of condition-specific activation or repression of transcriptional control elements are currently annotated in ENCODE. Similarly, information from human fetal tissue, reproductive organs and primary cell types is limited. In addition, although many open chromatin regions have been mapped, the transcription factors that bind to these sequences are largely unknown, and little attention has been devoted to the analysis of repetitive sequences. Finally, although transcript heterogeneity and isoforms have been described in many cell types, full-length transcripts that represent the isoform structure of spliced exons and edits have been described for only a small number of cell types.

Thus, as part of ENCODE 4, considerable effort is being devoted to expanding the cell types and tissues analysed (see URLs in Supplementary Note 1) as well as mapping the binding regions for many more transcription factors and RNA-binding proteins. These efforts are largely focused in a few reference cell lines, with the hope that improved knowledge will help with imputation or predictions in other cell states^34^. Single-cell transcriptome capture agents^35^ and open chromatin assays^36^ are also being applied to increase our understanding of the cellular heterogeneity of different tissues and samples. These efforts will supplement the many related activities that are also being pursued by HCA, HuBMAP and others^37,38^. Extensive mapping efforts of all types will continue in both the human and mouse, and parallel efforts to map transcription factor binding sites are being pursued in the *Drosophlia* and *C. elegans* by the modERN Project^28^. Full-length transcript isoforms are being elucidated in different cell types using long-read sequencing technologies^39^. ENCODE will continue to work with other consortia, and the data from different groups and individual laboratories will need to be consolidated into a common repository.

Importantly, although very large numbers of noncoding elements have been defined, the functional annotation of ENCODE-identified elements is still in its infancy. High-throughput reporter-based assays^40^, CRISPR-based genome and epigenome editing methods^41^, and other high-throughput approaches are being used in the current phase of ENCODE to assess the functions of many thousands of elements and to relate those functional results to their biochemical signatures. These targeted functional assays, combined with the large-scale annotation of biochemical features, should further enhance the value of ENCODE data.

Through these and other efforts, it is expected that many more elements in the human genome will be identified across a variety of cell types and conditions, their activities will be revealed (often at the single-cell level), and their biological functions will be inferred more accurately. The development of a systems-wide understanding of function and integration with genetic information associated with human traits will greatly enhance our understanding of human biology and disease.

## Online content

Any methods, additional references, Nature Research reporting summaries, source data, extended data, supplementary information, acknowledgements, peer review information; details of author contributions and competing interests; and statements of data and code availability are available at 10.1038/s41586-020-2449-8.

## Supplementary information

Supplementary Information This file contains the full author list for The ENCODE Project Consortium, and Supplementary Note 1 (Useful URLs).
