# Author Correction: Perspectives on ENCODE

**DOI:** 10.1038/s41586-021-04213-8

**Published:** 2022-04-26

**Authors:** The ENCODE Project Consortium, Federi Bronwen co Abascal, Reyes Acosta, Nicholas J. Addleman, Jessika Adrian, Veena Afzal, Bronwen Aken, Rizi Ai, Jennifer A. Akiyama, Omar Al Jammal, Henry Amrhein, Stacie M. Anderson, Gregory R. Andrews, Igor Antoshechkin, Kristin G. Ardlie, Joel Armstrong, Matthew Astley, Budhaditya Banerjee, Amira A. Barkal, If H. A. Barnes, Iros Barozzi, Daniel Barrell, Gemma Barson, Daniel Bates, Ulugbek K. Baymuradov, Cassandra Bazile, Michael A. Beer, Samantha Beik, M. A. Bender, Ruth Bennett, Louis Philip Benoit Bouvrette, Bradley E. Bernstein, Andrew Berry, Anand Bhaskar, Alexandra Bignell, Steven M. Blue, David M. Bodine, Carles Boix, Nathan Boley, Tyler Borrman, Beatrice Borsari, Alan P. Boyle, Laurel A. Brandsmeier, Alessandra Breschi, Emery H. Bresnick, Jason A. Brooks, Michael Buckley, Christopher B. Burge, Rachel Byron, Eileen Cahill, Lingling Cai, Lulu Cao, Mark Carty, Rosa G. Castanon, Andres Castillo, Hassan Chaib, Esther T. Chan, Daniel R. Chee, Sora Chee, Hao Chen, Huaming Chen, Jia-Yu Chen, Songjie Chen, J. Michael Cherry, Surya B. Chhetri, Jyoti S. Choudhary, Jacqueline Chrast, Dongjun Chung, Declan Clarke, Neal A. L. Cody, Candice J. Coppola, Julie Coursen, Anthony M. D’Ippolito, Stephen Dalton, Cassidy Danyko, Claire Davidson, Jose Davila-Velderrain, Carrie A. Davis, Job Dekker, Alden Deran, Gilberto DeSalvo, Gloria Despacio-Reyes, Colin N. Dewey, Diane E. Dickel, Morgan Diegel, Mark Diekhans, Vishnu Dileep, Bo Ding, Sarah Djebali, Alexander Dobin, Daniel Dominguez, Sarah Donaldson, Jorg Drenkow, Timothy R. Dreszer, Yotam Drier, Michael O. Duff, Douglass Dunn, Catharine Eastman, Joseph R. Ecker, Matthew D. Edwards, Nicole El-Ali, Shaimae I. Elhajjajy, Keri Elkins, Andrew Emili, Charles B. Epstein, Rachel C. Evans, Iakes Ezkurdia, Kaili Fan, Peggy J. Farnham, Nina Farrell, Elise A. Feingold, Anne-Maud Ferreira, Katherine Fisher-Aylor, Stephen Fitzgerald, Paul Flicek, Chuan Sheng Foo, Kevin Fortier, Adam Frankish, Peter Freese, Shaliu Fu, Xiang-Dong Fu, Yu Fu, Yoko Fukuda-Yuzawa, Mariateresa Fulciniti, Alister P. W. Funnell, Idan Gabdank, Timur Galeev, Mingshi Gao, Carlos Garcia Giron, Tyler H. Garvin, Chelsea Anne Gelboin-Burkhart, Grigorios Georgolopoulos, Mark B. Gerstein, Belinda M. Giardine, David K. Gifford, David M. Gilbert, Daniel A. Gilchrist, Shawn Gillespie, Thomas R. Gingeras, Peng Gong, Alvaro Gonzalez, Jose M. Gonzalez, Peter Good, Alon Goren, David U. Gorkin, Brenton R. Graveley, Michael Gray, Jack F. Greenblatt, Ed Griffiths, Mark T. Groudine, Fabian Grubert, Mengting Gu, Roderic Guigó, Hongbo Guo, Yu Guo, Yuchun Guo, Gamze Gursoy, Maria Gutierrez-Arcelus, Jessica Halow, Ross C. Hardison, Matthew Hardy, Manoj Hariharan, Arif Harmanci, Anne Harrington, Jennifer L. Harrow, Tatsunori B. Hashimoto, Richard D. Hasz, Meital Hatan, Eric Haugen, James E. Hayes, Peng He, Yupeng He, Nastaran Heidari, David Hendrickson, Elisabeth F. Heuston, Jason A. Hilton, Benjamin C. Hitz, Abigail Hochman, Cory Holgren, Lei Hou, Shuyu Hou, Yun-Hua E. Hsiao, Shanna Hsu, Hui Huang, Tim J. Hubbard, Jack Huey, Timothy R. Hughes, Toby Hunt, Sean Ibarrientos, Robbyn Issner, Mineo Iwata, Osagie Izuogu, Tommi Jaakkola, Nader Jameel, Camden Jansen, Lixia Jiang, Peng Jiang, Audra Johnson, Rory Johnson, Irwin Jungreis, Madhura Kadaba, Maya Kasowski, Mary Kasparian, Momoe Kato, Rajinder Kaul, Trupti Kawli, Michael Kay, Judith C. Keen, Sunduz Keles, Cheryl A. Keller, David Kelley, Manolis Kellis, Pouya Kheradpour, Daniel Sunwook Kim, Anthony Kirilusha, Robert J. Klein, Birgit Knoechel, Samantha Kuan, Michael J. Kulik, Sushant Kumar, Anshul Kundaje, Tanya Kutyavin, Julien Lagarde, Bryan R. Lajoie, Nicole J. Lambert, John Lazar, Ah Young Lee, Donghoon Lee, Elizabeth Lee, Jin Wook Lee, Kristen Lee, Christina S. Leslie, Shawn Levy, Bin Li, Hairi Li, Nan Li, Shantao Li, Xiangrui Li, Yang I. Li, Ying Li, Yining Li, Yue Li, Jin Lian, Maxwell W. Libbrecht, Shin Lin, Yiing Lin, Dianbo Liu, Jason Liu, Peng Liu, Tingting Liu, X. Shirley Liu, Yan Liu, Yaping Liu, Maria Long, Shaoke Lou, Jane Loveland, Aiping Lu, Yuheng Lu, Eric Lécuyer, Lijia Ma, Mark Mackiewicz, Brandon J. Mannion, Michael Mannstadt, Deepa Manthravadi, Georgi K. Marinov, Fergal J. Martin, Eugenio Mattei, Kenneth McCue, Megan McEown, Graham McVicker, Sarah K. Meadows, Alex Meissner, Eric M. Mendenhall, Christopher L. Messer, Wouter Meuleman, Clifford Meyer, Steve Miller, Matthew G. Milton, Tejaswini Mishra, Dianna E. Moore, Helen M. Moore, Jill E. Moore, Samuel H. Moore, Jennifer Moran, Ali Mortazavi, Jonathan M. Mudge, Nikhil Munshi, Rabi Murad, Richard M. Myers, Vivek Nandakumar, Preetha Nandi, Anil M. Narasimha, Aditi K. Narayanan, Hannah Naughton, Fabio C. P. Navarro, Patrick Navas, Jurijs Nazarovs, Jemma Nelson, Shane Neph, Fidencio Jun Neri, Joseph R. Nery, Amy R. Nesmith, J. Scott Newberry, Kimberly M. Newberry, Vu Ngo, Rosy Nguyen, Thai B. Nguyen, Tung Nguyen, Andrew Nishida, William S. Noble, Catherine S. Novak, Eva Maria Novoa, Briana Nuñez, Charles W. O’Donnell, Sara Olson, Kathrina C. Onate, Ericka Otterman, Hakan Ozadam, Michael Pagan, Tsultrim Palden, Xinghua Pan, Yongjin Park, E. Christopher Partridge, Benedict Paten, Florencia Pauli-Behn, Michael J. Pazin, Baikang Pei, Len A. Pennacchio, Alexander R. Perez, Emily H. Perry, Dmitri D. Pervouchine, Nishigandha N. Phalke, Quan Pham, Doug H. Phanstiel, Ingrid Plajzer-Frick, Gabriel A. Pratt, Henry E. Pratt, Sebastian Preissl, Jonathan K. Pritchard, Yuri Pritykin, Michael J. Purcaro, Qian Qin, Giovanni Quinones-Valdez, Ines Rabano, Ernest Radovani, Anil Raj, Nisha Rajagopal, Oren Ram, Lucia Ramirez, Ricardo N. Ramirez, Dylan Rausch, Soumya Raychaudhuri, Joseph Raymond, Rozita Razavi, Timothy E. Reddy, Thomas M. Reimonn, Bing Ren, Alexandre Reymond, Alex Reynolds, Suhn K. Rhie, John Rinn, Miguel Rivera, Juan Carlos Rivera-Mulia, Brian Roberts, Jose Manuel Rodriguez, Joel Rozowsky, Russell Ryan, Eric Rynes, Denis N. Salins, Richard Sandstrom, Takayo Sasaki, Shashank Sathe, Daniel Savic, Alexandra Scavelli, Jonathan Scheiman, Christoph Schlaffner, Jeffery A. Schloss, Frank W. Schmitges, Lei Hoon See, Anurag Sethi, Manu Setty, Anthony Shafer, Shuo Shan, Eilon Sharon, Quan Shen, Yin Shen, Richard I. Sherwood, Minyi Shi, Sunyoung Shin, Noam Shoresh, Kyle Siebenthall, Cristina Sisu, Teri Slifer, Cricket A. Sloan, Anna Smith, Valentina Snetkova, Michael P. Snyder, Damek V. Spacek, Sharanya Srinivasan, Rohith Srivas, George Stamatoyannopoulos, John A. Stamatoyannopoulos, Rebecca Stanton, Dave Steffan, Sandra Stehling-Sun, J. Seth Strattan, Amanda Su, Balaji Sundararaman, Marie-Marthe Suner, Tahin Syed, Matt Szynkarek, Forrest Y. Tanaka, Danielle Tenen, Mingxiang Teng, Jeffrey A. Thomas, Dave Toffey, Michael L. Tress, Diane E. Trout, Gosia Trynka, Junko Tsuji, Sean A. Upchurch, Oana Ursu, Barbara Uszczynska-Ratajczak, Mia C. Uziel, Alfonso Valencia, Benjamin Van Biber, Arjan G. van der Velde, Eric L. Van Nostrand, Yekaterina Vaydylevich, Jesus Vazquez, Alec Victorsen, Jost Vielmetter, Jeff Vierstra, Axel Visel, Anna Vlasova, Christopher M. Vockley, Simona Volpi, Shinny Vong, Hao Wang, Mengchi Wang, Qin Wang, Ruth Wang, Tao Wang, Wei Wang, Xiaofeng Wang, Yanli Wang, Nathaniel K. Watson, Xintao Wei, Zhijie Wei, Hendrik Weisser, Sherman M. Weissman, Rene Welch, Robert E. Welikson, Zhiping Weng, Harm-Jan Westra, John W. Whitaker, Collin White, Kevin P. White, Andre Wildberg, Brian A. Williams, David Wine, Heather N. Witt, Barbara Wold, Maxim Wolf, James Wright, Rui Xiao, Xinshu Xiao, Jie Xu, Jinrui Xu, Koon-Kiu Yan, Yongqi Yan, Hongbo Yang, Xinqiong Yang, Yi-Wen Yang, Galip Gürkan Yardımcı, Brian A. Yee, Gene W. Yeo, Taylor Young, Tianxiong Yu, Feng Yue, Chris Zaleski, Chongzhi Zang, Haoyang Zeng, Weihua Zeng, Daniel R. Zerbino, Jie Zhai, Lijun Zhan, Ye Zhan, Bo Zhang, Jialing Zhang, Jing Zhang, Kai Zhang, Lijun Zhang, Peng Zhang, Qi Zhang, Xiao-Ou Zhang, Yanxiao Zhang, Zhizhuo Zhang, Yuan Zhao, Ye Zheng, Guoqing Zhong, Xiao-Qiao Zhou, Yun Zhu, Jared Zimmerman, Michael P. Snyder, Thomas R. Gingeras, Jill E. Moore, Zhiping Weng, Mark B. Gerstein, Bing Ren, Ross C. Hardison, John A. Stamatoyannopoulos, Brenton R. Graveley, Elise A. Feingold, Michael J. Pazin, Michael Pagan, Daniel A. Gilchrist, Benjamin C. Hitz, J. Michael Cherry, Bradley E. Bernstein, Eric M. Mendenhall, Daniel R. Zerbino, Adam Frankish, Paul Flicek, Richard M. Myers

**Affiliations:** 1 Department of Genetics, School of Medicine, Stanford University, Palo Alto, CA USA; 2 Cardiovascular Institute, Stanford School of Medicine, Stanford, CA USA; 3 Functional Genomics, Cold Spring Harbor Laboratory, Cold Spring Harbor, NY USA; 4 University of Massachusetts Medical School, Program in Bioinformatics and Integrative Biology, Worcester, MA USA; 5 Department of Thoracic Surgery, Clinical Translational Research Center, Shanghai Pulmonary Hospital, The School of Life Sciences and Technology, Tongji University, Shanghai, China; 6 Bioinformatics Program, Boston University, Boston, MA USA; 7 Yale University, New Haven, CT USA; 8 Ludwig Institute for Cancer Research, University of California, San Diego, La Jolla, CA USA; 9 Center for Epigenomics, University of California, San Diego, La Jolla, CA USA; 10 Department of Biochemistry and Molecular Biology, The Pennsylvania State University, University Park, PA USA; 11 Altius Institute for Biomedical Sciences, Seattle, WA USA; 12 Department of Genome Sciences, University of Washington, Seattle, WA USA; 13 Department of Medicine, University of Washington, Seattle, WA USA; 14 Department of Genetics and Genome Sciences, Institute for Systems Genomics, UConn Health, Farmington, CT USA; 15 National Human Genome Research Institute, National Institutes of Health, Bethesda, MD USA; 16 Broad Institute and Department of Pathology, Massachusetts General Hospital and Harvard Medical School, Boston, MA USA; 17 Biological Sciences, University of Alabama in Huntsville, Huntsville, AL USA; 18 HudsonAlpha Institute for Biotechnology, Huntsville, AL USA; 19 European Molecular Biology Laboratory, European Bioinformatics Institute, Wellcome Genome Campus, Cambridge, UK; 20 The Broad Institute of Harvard and MIT, Cambridge, MA USA; 21 MGH, Boston, MA USA; 22 Dana-Farber Cancer Institute, Boston, MA USA; 23 Harvard Medical School, Boston, MA USA; 24 Boston Children’s Hospital, Boston, MA USA; 25 Harvard University, Cambridge, MA USA; 26 Computer Science and Artificial Intelligence Laboratory, Massachusetts Institute of Technology, Cambridge, MA USA; 27 Max Planck Institute for Molecular Genetics, Department of Genome Regulation, Berlin, Germany; 28 University of Colorado Boulder, Boulder, CO USA; 29 Bioinformatics and Genomics Program, Centre for Genomic Regulation (CRG), The Barcelona Institute of Science and Technology and Universitat Pompeu Fabra, Barcelona, Spain; 30 IRSD, Université de Toulouse, INSERM, INRA, ENVT, UPS, U1220, CHU Purpan, CS60039 Toulouse, France; 31 Skolkovo Institute for Science and Technology, Moscow, Russia; 32 Functional Genomics, Cold Spring Harbor Laboratory, Woodbury, NY USA; 33 Department of Clinical Research, University of Bern, Bern, Switzerland; 34 International Institute of Molecular and Cell Biology, Warsaw, Poland; 35 Department of Biology, Massachusetts Institute of Technology, Cambridge, MA USA; 36 Department of Cellular and Molecular Medicine, Institute for Genomic Medicine, Stem Cell Program, Sanford Consortium for Regenerative Medicine, University of California, San Diego, La Jolla, CA USA; 37 Département de Biochimie et Médecine Moléculaire, Université de Montréal, Montréal, Quebec, Canada; 38 Division of Experimental Medicine, McGill University, Quebec, Canada; 39 Institut de Recherches Cliniques de Montréal (IRCM), Montréal, Quebec Canada; 40 Program in Computational and Systems Biology, Massachusetts Institute of Technology, Cambridge, MA USA; 41 Department of Cellular and Molecular Medicine, Institute of Genomic Medicine, University of California, San Diego, San Diego, CA USA; 42 Department of Pharmaceutical Sciences, St. Jude Children’s Research Hospital, Memphis, TN USA; 43 Division of Biology, California Institute of Technology, Pasadena, CA USA; 44 National Human Genome Research Institute., National Institutes of Health, Bethesda, MD USA; 45 Department of Biostatistics and Bioinformatics, Duke University, Durham, NC USA; 46 Center for Genomic and Computational Biology, Duke University, Durham, NC USA; 47 Biological Sciences, University of California, Irvine, Irvine, CA USA; 48 Salk Institute for Biological Studies, La Jolla, CA USA; 49 Environmental Genomics and Systems Biology Division, Lawrence Berkeley National Laboratory, Berkeley, CA USA; 50 Department of Chemistry and Biochemistry, Department of Cellular and Molecular Medicine, UC San Diego, La Jolla, CA USA; 51 Institute for Human Genetics, Department of Neurology, University of California, San Francisco, San Francisco, CA USA; 52 Department of Biochemistry and Molecular Genetics, Northwestern University Feinberg School of Medicine, Chicago, IL USA; 53 Penn State Health Milton S. Hershey Medical Center, Hershey, PA USA; 54 US Department of Energy Joint Genome Institute, Lawrence Berkeley National Laboratory, Berkeley, CA USA; 55 School of Natural Sciences, University of California, Merced, Merced, CA USA; 56 Comparative Biochemistry Program, University of California, Berkeley, CA USA; 57 Howard Hughes Medical Institute, The Salk Institute for Biological Studies, La Jolla, CA USA; 58 Department of Computational Medicine and Bioinformatics, University of Michigan, Ann Arbor, MI USA; 59 Department of Human Genetics, University of Michigan, Ann Arbor, MI USA; 60 Department of Molecular and Cellular Physiology, School of Medicine, Stanford University, Palo Alto, CA USA; 61 Terrence Donnelly Centre for Cellular and Biomolecular Research, University of Toronto, Toronto, Ontario Canada; 62 Department of Genetics, School of Medicine, Yale University, New Haven, CT USA; 63 Department of Biochemistry and Molecular Medicine, Norris Comprehensive Cancer Center, Keck School of Medicine, University of Southern California, Los Angeles, CA USA; 64 Department of Radiation Oncology, School of Medicine, Stanford University, Palo Alto, CA USA; 65 Department of Human Genetics, Institute for Genomics and Systems Biology, The University of Chicago, Chicago, IL USA; 66 Division of General Surgery, Section of Transplant Surgery, School of Medicine, Washington University, St. Louis, MO USA; 67 Department of Biochemistry and Molecular Biology, School of Basic Medical Sciences, Southern Medical University, Guangzhou, China; 68 Guangdong Provincial Key Laboratory of Single Cell Technology and Application, Guangzhou, China; 69 Department of Cell Biology & Physiology, University of North Carolina at Chapel Hill, Chapel Hill, NC USA; 70 Thurston Arthritis Research Center, University of North Carolina at Chapel Hill, Chapel Hill, NC USA; 71 Department of Molecular Genetics, University of Toronto, Toronto, Ontario Canada; 72 School of Medicine, Jiangsu University, Zhenjiang, China; 73 Department of Molecular Genetics, Donnelly Centre, University of Toronto, Toronto, Ontario Canada; 74 Tempus Labs, Chicago, IL USA; 75 Department of Medicine, University of Washington., Seattle, WA USA; 76 Fred Hutchinson Cancer Research Center, Seattle, WA USA; 77 HHMI and Program in Systems Biology, University of Massachusetts Medical School, Albert Sherman Center, Worcester, MA USA; 78 University of Massachusetts Amherst, Amherst, MA USA; 79 Institute for Infocomm Research, Singapore, Singapore; 80 Simon Fraser University, Burnaby, British Columbia Canada; 81 Center for Functional Cancer Epigenetics, Dana-Farber Cancer Institute, Boston, MA USA; 82 Department of Data Sciences, Dana-Farber Cancer Institute and Harvard T.H. Chan School of Public Health, Boston, MA USA; 83 Center for Public Health Genomics, University of Virginia, Charlottesville, VA USA; 84 Molecular Pathology Unit & Cancer Center, Boston, MA USA; 85 Department of Biostatistics and Bioinformatics, Moffitt Cancer Center, Tampa, FL USA; 86 Institute for Stem Cell Biology and Regenerative Medicine, Stanford University, Stanford, CA USA; 87 Department of Medicine, Brigham and Women’s Hospital and Harvard Medical School, Boston, MA USA; 88 Department of Statistics, Medical Sciences Center, Madison, WI USA; 89 Department of Biostatistics and Medical Informatics, Madison, WI USA; 90 Department of Mathematical Sciences, University of Texas at Dallas, Richardson, TX USA; 91 Department of Statistics, University of Nebraska-Lincoln, Lincoln, NE USA; 92 Department of Biomedical Informatics, The Ohio State University, Columbus, OH USA; 93 Department of Cell and Regenerative Biology, UW-Madison Blood Research Program, Carbone Cancer Center, University of Wisconsin School of Medicine and Public Health, University of Wisconsin, Madison, WI USA; 94 Department of Genetics and Genomic Sciences, Icahn School of Medicine at Mount Sinai, New York, NY USA; 95 Wellcome Sanger Institute, Cambridge, UK; 96 Program in Computational Biology, Memorial Sloan Kettering Cancer Center, New York, NY USA; 97 610 Charles E. Young Drive S, Terasaki Life Sciences Building, Room 2000E, Los Angeles, CA USA; 98 McKusick-Nathans Institute of Genetic Medicine, Johns Hopkins University, Baltimore, MD USA; 99 Department of Biomedical Engineering, Johns Hopkins University, Baltimore, MD USA; 100 University of California, Santa Cruz, Santa Cruz, CA USA; 101 Center for Integrative Genomics, University of Lausanne, Lausanne, Switzerland; 102 Centro Nacional de Investigaciones Cardiovasculares (CNIC) and CIBER de Enfermedades Cardiovasculares (CIBERCV), Madrid, Spain; 103 Spanish National Cancer Research Centre (CNIO), Madrid, Spain; 104 Brunel University London, London, UK; 105 King’s College London, Guy’s Hospital, London, UK; 106 ELIXIR Hub, Wellcome Genome Campus, Cambridge, UK; 107 Institute of Cancer Research, London, UK; 108 Department of Biological Science, Florida State University, Tallahassee, FL USA; 109 Department of Biochemistry, Molecular Biology and Biophysics, University of Minnesota Medical School, Minneapolis, MN USA; 110 Center for Vaccines and Immunology University of Georgia, Athens, GA USA; 111 Center for Molecular Medicine and Department of Biochemistry and Molecular Biology, University of Georgia, Athens, GA USA; 112 Gift of Life Donor Program, Philadelphia, PA USA; 113 American Society for Radiation Oncology, Arlington, VA USA; 114 National Cancer Institute, National Institutes of Health, Bethesda, MD USA; 115 Leidos Biomedical, Inc, Frederick, MD USA; 116 National Disease Research Interchange (NDRI), Philadelphia, PA USA; 117 4407 Puller Drive, Kensington, MD USA; 118 Department of Chemistry and Biochemistry, University of California, San Diego, La Jolla, CA USA; 119 Program in Computational Biology and Bioinformatics, Yale University, New Haven, CT USA

Correction to: *Nature* 10.1038/s41586-020-2449-8published online 29 July 2020

In this Article, the authors Rizi Ai (Department of Chemistry and Biochemistry, University of California, San Diego, La Jolla, CA, USA) and Shantao Li (Program in Computational Biology and Bioinformatics, Yale University, New Haven, CT, USA) were mistakenly omitted from the ENCODE Project Consortium author list. The original Article has been corrected online.

