# Supplementary material for: Perspectives on ENCODE
Source: Nature. 2020 Jul 29;583(7818):693–8. doi: 10.1038/s41586-020-2449-8 (PMC7410827; doi:10.1038/s41586-020-2449-8)
Supplement: Supplementary file 1 — This file contains the full author list for The ENCODE Project Consortium, and Supplementary Note 1 (Useful URLs). [file 41586_2020_2449_MOESM1_ESM.pdf]

nature

https://doi.org/10.1038/s41586-020-2449-8

## Supplementary information

# Perspectives on ENCODE

In the format provided by the authors and unedited

The ENCODE Project Consortium, Michael P. Snyder✉, Thomas R. Gingeras, Jill E. Moore, Zhiping Weng, Mark B. Gerstein, Bing Ren, Ross C. Hardison, John A. Stamatoyannopoulos, Brenton R. Graveley, Elise A. Feingold, Michael J. Pazin, Michael Pagan, Daniel A. Gilchrist, Benjamin C. Hitz, J. Michael Cherry, Bradley E. Bernstein, Eric M. Mendenhall, Daniel R. Zerbino, Adam Frankish, Paul Flicek & Richard M. Myers

# Supplementary Information

# Perspectives on ENCODE


**ENCODE Perspective authors**

The ENCODE Project Consortium[#], Michael P. Snyder[1,2], Thomas R. Gingeras[3], Jill E. Moore[4], Zhiping Weng[4,5,6], Mark B. Gerstein[7], Bing Ren[8,9], Ross C. Hardison[10], John A. Stamatoyannopoulos[11,12,13], Brenton R. Graveley[14], Elise A. Feingold[15], Michael J. Pazin[15], Michael Pagan[15], Daniel A. Gilchrist[15], Benjamin C. Hitz[1], J. Michael Cherry[1], Bradley E. Bernstein[16], Eric M. Mendenhall[17,18], Daniel R. Zerbino[19], Adam Frankish[19], Paul Flicek[19], Richard M. Myers[18]

**The ENCODE Project Consortium**

**The Broad Institute of Harvard and MIT (data production and analysis)**

Charles B. Epstein[20], Noam Shoresh[20], Robbyn Issner[20], Shawn Gillespie[21], Dylan Rausch[21], Joseph Raymond[20], Shanna Hsu[20], Danielle Tenen[20], Oren Ram[20], Alon Goren[20], Russell Ryan[21], Mariateresa Fulciniti[22], David Hendrickson[20], Jonathan Scheiman[23], Birgit Knoechel[22,24], David Kelley[25], Samantha Beik[20], Yotam Drier[21], Carles Boix[26], Wouter Meuleman[11], Pouya Kheradpour[26], Nina Farrell[20], Meital Hatan[20], David Wine[20], Mia C. Uziel[20], Kristin G. Ardlie[20], Michael Mannstadt[21], Nikhil Munshi[22], Miguel Rivera[21], Alex Meissner[27], Manolis Kellis[20,26], John Rinn[28], Bradley E. Bernstein[16]

**Cold Spring Harbor, Centre for Genomic Regulation (CRG), The Barcelona Institute of Science and Technology and Universitat Pompeu Fabra (data production and analysis)**

Carrie A. Davis[3], Alexander Dobin[3], Alessandra Breschi[29], Sarah Djebali[29,30], Chris Zaleski[3], Dmitri D. Pervouchine[29,31], Anna Vlasova[29], Jorg Drenkow[32], Julien Lagarde[29], Rory Johnson[29,33], Barbara Uszczynska-Ratajczak[29,34], Alexandra Scavelli[3], Cassidy Danyko[3], Lei Hoon See[3], Roderic Guigó[29], Thomas R. Gingeras[3]

**UConn Health, UCSD, Massachusetts Institute of Technology, and Institut de Recherches Cliniques de Montréal (IRCM) (data production and analysis)**

Cassandra Bazile[35], Steven M. Blue[36], Eric L. Van Nostrand[36], Louis Philip Benoit Bouvrette[37,38,39], Daniel Dominguez[35], Peter Freese[40], Jia-Yu Chen[41], Neal A.L. Cody[37,38,39], Gabriel A. Pratt[36], Michael O. Duff[14], Sara Olson[14], Xiaofeng Wang[37,38,39], Keri Elkins[36], Balaji Sundararaman[36], Xintao Wei[14], Chelsea Anne Gelboin-Burkhart[36], Rui Xiao[41], Abigail Hochman[35], Lijun Zhan[14], Nicole J. Lambert[35], Hairi Li[41], Thai B. Nguyen[36], Tsultrim Palden[35], Ines Rabano[36], Shashank Sathe[36], Rebecca Stanton[36], Amanda Su[35], Ruth Wang[36], Brian A. Yee[36], Xiang-Dong Fu[41], Eric Lécuyer[37,38,39], Christopher B. Burge[35], Gene W. Yeo[36], Brenton R. Graveley[14]

**HudsonAlpha Institute for Biotechnology, California Institute of Technology, The Pennsylvania State University, National Human Genome Research Institute, University of Alabama in Huntsville, Duke University, University of California Irvine (data production and analysis)**

Mark Mackiewicz[18], Florencia Pauli-Behn[18], E. Christopher Partridge[18], Daniel Savic[42], Brian Roberts[18], Kimberly M. Newberry[18], Laurel A. Brandsmeier[18], Sarah K. Meadows[18], Rosy Nguyen[18], Amy R. Nesmith[18], Dianna E. Moore[18], Christopher L. Messer[18], Megan McEown[18], Rachel C. Evans[18], J Scott Newberry[18], Collin White[18], Shawn Levy[18], Barbara Wold[43], Brian A. Williams[43], Diane E. Trout[43], Gilberto DeSalvo[43], Kenneth McCue[43], Peng He[43], Katherine Fisher-Aylor[43], Sean A. Upchurch[43], Henry Amrhein[43], Georgi K. Marinov[43], Jost Vielmetter[43], Anthony Kirilusha[43], Igor Antoshechkin[43], Ross C. Hardison[10], Cheryl A. Keller[10], Belinda M. Giardine[10], Maria Long[10], David M. Bodine[44], Elisabeth F. Heuston[44], Stacie M. Anderson[44], Eric M. Mendenhall[17,18], Surya B. Chhetri[17,18], Candice J. Coppola[17,18], Timothy E. Reddy[45,46], Anthony M. D'Ippolito[46], Christopher M. Vockley[20], Ali Mortazavi[47], Rabi Murad[47], Weihua Zeng[47], Camden Jansen[47], Ricardo N. Ramirez[47], Nicole El-Ali[47], Richard M. Myers[18]

**University of California, San Diego, Salk Institute for Biological Studies, Lawrence Berkeley National Laboratory, UC San Diego, Howard Hughes Medical Institute (data production and analysis)**

David U. Gorkin[8,9], Yupeng He[48], Iros Barozzi[49], Andre Wildberg[50], Jennifer A. Akiyama[49], Rosa G. Castanon[48], Sora Chee[8], Huaming Chen[48], Bo Ding[50], Yoko Fukuda-Yuzawa[49], Tyler H. Garvin[49], Manoj Hariharan[48], Anne Harrington[49], Hui Huang[8], Momoe Kato[49], Samantha Kuan[8], Ah Young Lee[8], Elizabeth Lee[49], Bin Li[8], Nan Li[50], Brandon J. Mannion[49], Joseph R. Nery[48], Vu Ngo[50], Tung Nguyen[50], Quan Pham[49], Catherine S. Novak[49], Ingrid Plajzer-Frick[49], Sebastian Preissl[8], Yin Shen[8,51], Mengchi Wang[50], Tao Wang[50], John W. Whitaker[50], Yanxiao Zhang[8], Kai

Zhang[50], Yuan Zhao[8], Yun Zhu[50], Feng Yue[52,53], Veena Afzal[49], Diane E. Dickel[49], Valentina Snetkova[49], Wei Wang[50], Axel Visel[49,54,55], Len A. Pennacchio[49,54,56], Joseph R. Ecker[48,57], Bing Ren[8,9]

**Stanford University, The University of Chicago, University of Southern California, University of Toronto, Yale University (data production and analysis)**

Jessika Adrian[1], Trupti Kawli[1], Nicholas J. Addleman[1], Alan P. Boyle[58,59], Lulu Cao[1], Hassan Chaib[1], Songjie Chen[1], Catharine Eastman[60], Andrew Emili[61], Peng Gong[62], Fabian Grubert[1], Yu Guo[63], Hongbo Guo[61], Nastaran Heidari[1,64], Cory Holgren[65], Nader Jameel[65], Lixia Jiang[1], Madhura Kadaba[65], Maya Kasowski[1], Mary Kasparian[65], Yining Li[1], Jin Lian[62], Yiing Lin[66], Shin Lin[1], Lijia Ma[65], Matthew G. Milton[65], Tejaswini Mishra[1], Jennifer Moran[65], Anil M. Narasimha[1], Xinghua Pan[62,67,68], Doug H. Phanstiel[69,70], Ernest Radovani[61], Lucia Ramirez[1], Rozita Razavi[71], Suhn K. Rhie[63], Denis N. Salins[1], Frank W. Schmitges[71], Quan Shen[62,72], Minyi Shi[1], Teri Slifer[1], Damek V. Spacek[1], Rohith Srivas[1], Dave Steffan[65], Matt Szynkarek[65], Dave Toffey[65], Alec Victorsen[65], Nathaniel K. Watson[1], Heather N. Witt[63], Xinqiong Yang[1], Jie Zhai[1], Jialing Zhang[62], Guoqing Zhong[61], Sherman M. Weissman[62], Jack F. Greenblatt[61,71], Timothy R. Hughes[61,73], Peggy J. Farnham[63], Kevin P. White[74], Michael P. Snyder[1,2]

**Altius Institute for Biomedical Sciences, University of Washington, Fred Hutchinson Cancer Research Center, University of Massachusetts Medical School, Howard Hughes Medical Institute (data production and analysis)**

John A. Stamatoyannopoulos[11,12,13], Rajinder Kaul[11,13], Jessica Halow[11], Richard Sandstrom[11], Michael Buckley[11], Jeff Vierstra[11], Wouter Meuleman[11], Eric Haugen[11], Shane Neph[11], Andrew Nishida[11], Alex Reynolds[11], Eric Rynes[11], Audra Johnson[11], Jemma Nelson[11], Alister P. W. Funnell[11], Vivek Nandakumar[11], Kyle Siebenthall[11], Hao Wang[11], Yongqi Yan[11], Reyes Acosta[11], Kristen Lee[11], Ericka Otterman[11], Benjamin Van Biber[11], Mineo Iwata[11], Tanya Kutyavin[11], Sandra Stehling-Sun[11], Robert E. Welikson[11], Andres Castillo[11], Grigorios Georgolopoulos[11], Sean Ibarrientos[11], Fidencio Jun Neri[11], Anthony Shafer[11], Shinny Vong[11], Daniel Bates[11], Morgan Diegel[11], Douglass Dunn[11], John Lazar[11], Daniel R. Chee[11], George Stamatoyannopoulos[75], Patrick Navas[11], M. A. Bender[76], Mark T. Groudine[76], Rachel Byron[76], Ye Zhan[77], Hakan Ozadam[77], Bryan R. Lajoie[77], Job Dekker[77]

**Data Coordination Center at Stanford (data coordination center)**

J. Michael Cherry[1], Benjamin C. Hitz[1], Cricket A. Sloan[1], Ulugbek K. Baymuradov[1], Esther T.

Chan[1], Timothy R. Dreszer[1], Idan Gabdank[1], Jason A. Hilton[1], Aditi K. Narayanan[1], Kathrina C. Onate[1], J. Seth Strattan[1], Forrest Y. Tanaka[1]

**University of Massachusetts Medical School, Yale University, Stanford University, Centre for Genomic Regulation (CRG), The Barcelona Institute of Science and Technology and Universitat Pompeu Fabra, University of Washington, Dana-Farber Cancer Institute, Harvard School of Public Health, Massachusetts Institute of Technology (data analysis center)**

Jill E. Moore[4], Michael J. Purcaro[4], Henry E. Pratt[4], Gregory R. Andrews[4], Tyler Borrman[4], Jason A. Brooks[4], Hao Chen[4,6], Shaimae I. Elhajjajy[4], Kaili Fan[4], Kevin Fortier[4], Mingshi Gao[4], Jack Huey[4], Eugenio Mattei[4], Nishigandha N. Phalke[4], Thomas M. Reimonn[4], Shuo Shan[4], Junko Tsuji[4], Arjan G. van der Velde[4,6], Taylor Young[4], Xiao-Ou Zhang[4], Tianxiong Yu[5], Peng Zhang[5], Lingling Cai[5], Xiangrui Li[5], Ying Li[5], Zhijie Wei[5], Shuyu Hou[5], Aiping Lu[5], Yan Liu[5], Yu Fu[4,78], Shaliu Fu[5], Qin Wang[5], Joel Rozowsky[7], Jing Zhang[7], Koon-Kiu Yan[7], Mengting Gu[7], Donghoon Lee[7], Shaoke Lou[7], Anurag Sethi[7], Sushant Kumar[7], Timur Galeev[7], Arif Harmanci[7], Gamze Gursoy[7], Jason Liu[7], Jinrui Xu[7], Fabio C.P. Navarro[7], Nathan Boley[1], Daniel Sunwook Kim[1], Jin Wook Lee[1], Chuan Sheng Foo[79], Oana Ursu[1], Sarah Djebali[29,30], Dmitri D. Pervouchine[29,31], Anna Vlasova[29], Beatrice Borsari[29], Maxwell W. Libbrecht[80], Galip Gürkan Yardımcı[12], Peng Jiang[81,82], Clifford Meyer[81,82], Chongzhi Zang[81,82,83], Qian Qin[23,84], Mingxiang Teng[85], Jose Davila-Velderrain[26], Yue Li[26], Zhizhuo Zhang[26], Dianbo Liu[26], Carles Boix[26], Yongjin Park[26], Yaping Liu[26], Lei Hou[26], Manolis Kellis[20,26], X. Shirley Liu[81,82], William S. Noble[12], Roderic Guigó[29], Anshul Kundaje[1], Mark B. Gerstein[7], Zhiping Weng[4,5,6]

**Massachusetts Institute of Technology (computational algorithm development)**

Amira A. Barkal[86], Budhaditya Banerjee[87], Matthew D. Edwards[26], David K. Gifford[26], Yuchun Guo[26], Tatsunori B. Hashimoto[26], Tommi Jaakkola[26], Charles W. O'Donnell[26], Nisha Rajagopal[87], Richard I. Sherwood[87], Sharanya Srinivasan[87], Tahin Syed[26], Haoyang Zeng[26]

**University of Wisconsin–Madison, University of Nebraska-Lincoln, The Ohio State University, University of Wisconsin School of Medicine and Public Health (computational algorithm development)**

Ye Zheng[88], Peng Liu[89], Sunyoung Shin[90], Rene Welch[89], Jurijs Nazarovs[88], Qi Zhang[91], Dongjun Chung[92], Emery H. Bresnick[93], Colin N. Dewey[89], Sunduz Keles[88,89]

**Icahn School of Medicine at Mount Sinai, Brigham and Women's Hospital and Harvard Medical School, Memorial Sloan Kettering Cancer Center (computational algorithm development)**
James E. Hayes[94], Gosia Trynka[95], Alvaro Gonzalez[96], Harm-Jan Westra[87], Manu Setty[96], Maria Gutierrez-Arcelus[87], Yuheng Lu[96], Alexander R. Perez[96], Yuri Pritykin[96], Mark Carty[96], Christina S. Leslie[96], Soumya Raychaudhuri[87], Robert J. Klein[94]

**Stanford (computational algorithm development)**
Anand Bhaskar[1], Yang I. Li[1], Graham McVicker[48], Eilon Sharon[1], Anil Raj[1], Jonathan K. Pritchard[1]

**University of California, Los Angeles (computational algorithm development)**
Yun-Hua E. Hsiao[97], Giovanni Quinones-Valdez[97], Yi-Wen Yang[97], Xinshu Xiao[97]

**Johns Hopkins University (data analysis)**
Michael A. Beer[98,99]

**Pennsylvania State University/Northwestern University (data production and analysis)**
Yanli Wang[53], Hongbo Yang[53], Tingting Liu[53], Lijun Zhang[53], Jie Xu[53], Bo Zhang[53], Feng Yue[52,53]

**European Bioinformatics Institute (EMBL-EBI), Centre for Genomic Regulation (CRG), The Barcelona Institute of Science and Technology and Universitat Pompeu Fabra, ELIXIR Hub, King's College London, Guy's Hospital, Massachusetts Institute of Technology, Spanish National Cancer Research Centre (CNIO), University of Bern, University of California, Santa Cruz, University of Lausanne, Wellcome Sanger Institute, Yale University (gene annotation)**
Bronwen Aken[19], Joel Armstrong[100], Matthew Astley[95], If H.A. Barnes[19], Daniel Barrell[19], Gemma Barson[95], Ruth Bennett[19], Andrew Berry[19], Alexandra Bignell[19], Jacqueline Chrast[101], Declan Clarke[7], Claire Davidson[19], Alden Deran[100], Gloria Despacio-Reyes[95], Mark Diekhans[100], Sarah Donaldson[19], Iakes Ezkurdia[102], Anne-Maud Ferreira[101], Stephen Fitzgerald[95], Carlos Garcia Giron[19], Jose M. Gonzalez[19], Michael Gray[95], Ed Griffiths[95], Matthew Hardy[19], Toby Hunt[19], Rory Johnson[29,33], Irwin Jungreis[20,26], Michael Kay[19], Julien Lagarde[29], Jane Loveland[19], Deepa Manthravadi[95], Osagie Izuogu[19], Fergal J. Martin[19], Steve Miller[95], Jonathan M. Mudge[19], Eva Maria Novoa[26], Baikang Pei[7], Dmitri D. Pervouchine[29,31], Jose M. Rodrigez[103], Christoph

Schlaffner[95], Cristina Sisu[7,104], Marie-Marthe Suner[19], Michael L. Tress[103], Barbara Uszczynska-Ratajczak[29,34], Jesus Vazquez[102], Hendrik Weisser[95], Maxim Wolf[26], James Wright[95], Tim J. Hubbard[105], Jennifer L. Harrow[106], Alfonso Valencia[103], Federico Abascal[95], Manolis Kellis[20,26], Paul Flicek[19], Benedict Paten[100], Jyoti S. Choudhary[107], Mark B. Gerstein[7], Alexandre Reymond[101], Roderic Guigó[29], Adam Frankish[19]

**Florida State University and University of Georgia (data production and analysis)**
Juan Carlos Rivera-Mulia[108,109], Takayo Sasaki[108], Vishnu Dileep[108], Jared Zimmerman[108], Michael J. Kulik[110], Stephen Dalton[111], David M. Gilbert[108]

**European Bioinformatics Institute (EMBL-EBI) (genome annotation)**
Emily H. Perry[19], Daniel R. Zerbino[19], Paul Flicek[19]

**The Broad Institute of Harvard and MIT, Gift of Life Donor Program, American Society for Radiation Oncology, National Cancer Institute (NCI), Leidos Biomedical, Inc., National Disease Research Interchange (NDRI), National Human Genome Research Institute (NHGRI) (tissue sample preparation)**
Kristin G. Ardlie[20], Richard D. Hasz[112], Judith C. Keen[113], Helen M. Moore[114], Anna Smith[115], Jeffrey A. Thomas[116], Simona Volpi[15]

**National Human Genome Research Institute (Project Management)**
Xiao-Qiao Zhou[15], Hannah Naughton[15], Julie Coursen[15], Samuel H. Moore[15], Preetha Nandi[15], Omar Al Jammal[15], Yekaterina Vaydylevich[15], Peter Good[117], Jeffery A. Schloss[15], Briana Nuñez[15], Michael Pagan[15], Eileen Cahill[15], Daniel A. Gilchrist[15], Michael J. Pazin[15], Elise A. Feingold[15]
(*The role of the NHGRI Project Management Group in the preparation of this paper was limited to coordination and scientific management of the ENCODE consortium.*)

**Affiliations**
1) Department of Genetics, School of Medicine, Stanford University, Palo Alto, California 94305, USA.
2) Cardiovascular Institute, Stanford School of Medicine, Stanford CA 94305
3) Cold Spring Harbor Laboratory, Functional Genomics, 1 Bungtown Road, Cold Spring Harbor, New York 11742, USA

4) University of Massachusetts Medical School, Program in Bioinformatics and Integrative Biology, 368 Plantation St., Worcester, MA 01605, USA
5) Department of Thoracic Surgery, Clinical Translational Research Center, Shanghai Pulmonary Hospital, The School of Life Sciences and Technology, Tongji University, Shanghai 200092, China
6) Bioinformatics Program, Boston University, Boston, MA 02215, USA
7) Yale University, New Haven, Connecticut 06520-8047, USA
8) Ludwig Institute for Cancer Research, University of California, San Diego, 9500 Gilman Drive, La Jolla, CA 92093-0653
9) Center for Epigenomics, University of California, San Diego, 9500 Gilman Drive, La Jolla, CA 92093-0653
10) Department of Biochemistry and Molecular Biology, The Pennsylvania State University, 304 Wartik Laboratory, University Park PA 16802
11) Altius Institute for Biomedical Sciences, 2211 Elliott Avenue, Suite 410, Seattle, WA, 98121
12) Department of Genome Sciences, University of Washington, 3720 15th Ave NE Seattle, Washington 98195-5065, USA.
13) Department of Medicine, RR-512, Health Sciences Building, University of Washington, Box 356420, 1959 NE Pacific Street, Seattle, WA 98195-6420
14) Department of Genetics and Genome Sciences, Institute for Systems Genomics, UConn Health, Farmington, CT 06030, USA
15) National Human Genome Research Institute, National Institutes of Health, 6700B Rockledge Drive, Bethesda, MD 20817
16) Broad Institute and Department of Pathology, Massachusetts General Hospital and Harvard Medical School, Boston, MA 02114
17) Biological Sciences, University of Alabama in Huntsville, 301 Sparkman Drive, Huntsville AL 35899
18) HudsonAlpha Institute for Biotechnology, 601 Genome Way, Huntsville, AL 35806
19) European Molecular Biology Laboratory, European Bioinformatics Institute, Wellcome Genome Campus, Hinxton, Cambridge, CB10 1SD, United Kingdom
20) The Broad Institute of Harvard and MIT, 415 Main St, Cambridge, MA 02142
21) MGH, 55 Fruit St, Boston, MA 02114
22) Dana-Farber Cancer Institute, 450 Brookline Ave, Boston, MA 02215
23) Harvard Medical School, 25 Shattuck St, Boston, MA 02115
24) Boston Children's Hospital, 300 Longwood Ave, Boston, MA 02115

25) Harvard University, Massachusetts Hall, Cambridge, MA 02138
26) Computer Science and Artificial Intelligence Laboratory, Massachusetts Institute of Technology, Cambridge, MA 02139
27) Max Planck Institute for Molecular Genetics, Department of Genome Regulation, Ihnestr. 63-73, Berlin 14195 Germany
28) University of Colorado Boulder, Boulder CO 80301
29) Bioinformatics and Genomics Program, Centre for Genomic Regulation (CRG), The Barcelona Institute of Science and Technology and Universitat Pompeu Fabra, Dr. Aiguader 88, 08003 Barcelona, Catalonia, Spain
30) IRSD, Université de Toulouse, INSERM, INRA, ENVT, UPS, U1220, CHU Purpan, CS60039, 31024, Toulouse, France
31) Skolkovo Institute for Science and Technology, Bolshoy Boulevard 30, bld. 1 Moscow 121205, Russia
32) Cold Spring Harbor Laboratory, 500 Sunnyside Blvd, Woodbury NY 11797, USA
33) Department of Clinical Research, University of Bern, Murtenstrasse 35, 3010 Bern, Switzerland.
34) International Institute of Molecular and Cell Biology, Ks. Trojdena 4 02-109 Warsaw Poland
35) Department of Biology, Massachusetts Institute of Technology, Cambridge, MA 02142, USA
36) Department of Cellular and Molecular Medicine, Institute for Genomic Medicine, Stem Cell Program, Sanford Consortium for Regenerative Medicine, University of California, San Diego, La Jolla, CA 92037, USA
37) Département de Biochimie et Médecine Moléculaire, Université de Montréal, Montréal, QC H2W 1R7, Canada
38) Division of Experimental Medicine, McGill University, QC H2W 1R7, Canada
39) Institut de Recherches Cliniques de Montréal (IRCM), Montréal, QC H2W 1R7, Canada
40) Program in Computational and Systems Biology, Massachusetts Institute of Technology, Cambridge, MA 02142, USA
41) Department of Cellular and Molecular Medicine, Institute of Genomic Medicine, University of California at San Diego, San Diego, California 92093, USA
42) Department of Pharmaceutical Sciences, St. Jude Children’s Research Hospital, Memphis, TN 38105, USA
43) Division of Biology 156-29, 1200 E. California Blvd, California Institute of Technology, Pasadena CA 91125

44) National Human Genome Research Institute, National Institutes of Health, Building 49, Room 4A04, 40 Convent Drive, Bethesda, MD 20892
45) Department of Biostatistics and Bioinformatics, Duke University, Durham, NC 27708, USA
46) Center for Genomic and Computational Biology, Duke University, Durham, NC 27708, USA
47) 2218 Biological Sciences III, University of California Irvine, Irvine CA 92697-2300
48) Salk Institute for Biological Studies, 10010 N Torrey Pines Rd, La Jolla, CA 92037
49) Environmental Genomics and Systems Biology Division, Lawrence Berkeley National Laboratory, 1 Cyclotron Road, Berkeley, CA 94720, USA
50) Department of Chemistry and Biochemistry, Department of Cellular and Molecular Medicine, UC San Diego, 9500 Gilman Drive, La Jolla, CA 92093-0359
51) Institute for Human Genetics, Department of Neurology, University of California, San Francisco, San Francisco, California 94143, USA
52) Department of Biochemistry and Molecular Genetics, Northwestern University Feinberg School of Medicine, Chicago, IL 60611, USA.
53) Penn State Health Milton S. Hershey Medical Center, 500 University Dr., Hershey, PA 17033, USA
54) U.S. Department of Energy Joint Genome Institute, Lawrence Berkeley National Laboratory, 1 Cyclotron Road, Berkeley, CA 94720, USA
55) School of Natural Sciences, University of California, Merced, Merced, California, USA
56) Comparative Biochemistry Program, University of California, Berkeley, CA 94720, USA
57) Howard Hughes Medical Institute, The Salk Institute for Biological Studies, 10010 N Torrey Pines Rd, La Jolla, CA 92037
58) Department of Computational Medicine and Bioinformatics, University of Michigan, Ann Arbor, MI 48109, USA.
59) Department of Human Genetics, University of Michigan, Ann Arbor, MI 48109, USA.
60) Department of Molecular and Cellular Physiology, School of Medicine, Stanford University, Palo Alto, California 94305, USA.
61) Terrence Donnelly Centre for Cellular and Biomolecular Research, University of Toronto, Toronto, Ontario M5S 3E1, Canada.
62) Department of Genetics, School of Medicine, Yale University, New Haven, Connecticut 06520, USA.
63) Department of Biochemistry and Molecular Medicine, Norris Comprehensive Cancer Center, Keck School of Medicine, University of Southern California, Los Angeles, California 90089, USA.

64) Department of Radiation Oncology, School of Medicine, Stanford University, Palo Alto, California 94305, USA
65) Department of Human Genetics, Institute for Genomics and Systems Biology, The University of Chicago, Chicago, Illinois 60637, USA.
66) Division of General Surgery, Section of Transplant Surgery, School of Medicine, Washington University, St. Louis, Missouri 63110, USA.
67) Department of Biochemistry and Molecular Biology, School of Basic Medical Sciences, Southern Medical University, Guangzhou 510515, Guangzhou, China
68) Guangdong Provincial Key Laboratory of Single Cell Technology and Application, Guangzhou 510515, Guangdong, China
69) Department of Cell Biology & Physiology, University of North Carolina at Chapel Hill, Chapel Hill, NC 27599
70) Thurston Arthritis Research Center, University of North Carolina at Chapel Hill, Chapel Hill, NC 27599
71) Department of Molecular Genetics, University of Toronto, Toronto, Ontario M5S 1A8, Canada.
72) School of Medicine, Jiangsu University, Zhenjiang, Jiangsu 212013, China
73) Department of Molecular Genetics, Donnelly Centre, University of Toronto, Toronto, Ontario M5S 3E1, Canada
74) Tempus Labs, 600 W Chicago Ave. Ste 510, Chicago, IL 60654
75) Department of Medicine, University of Washington, K-240 Health Sciences Building, Box 357720, Seattle, WA 98195
76) Fred Hutchinson Cancer Research Center, 1100 Fairview ave. N, Seattle, WA 98109
77) HHMI and Program in Systems Biology, University of Massachusetts Medical School, 368 Plantation Street, Albert Sherman Center, Worcester, MA 01605
78) University of Massachusetts Amherst, 270 Stockbridge Rd, Amherst, MA 01003, USA
79) Institute for Infocomm Research, 1 Fusionopolis Way, #21-01 Connexis (South Tower), Singapore 138632, Singapore
80) Simon Fraser University, ASB 9971-8888 University Drive. Burnaby, British Columbia V5A 1S6, Canada
81) Center for Functional Cancer Epigenetics, Dana-Farber Cancer Institute, Boston, MA 02215
82) Department of Data Sciences, Dana-Farber Cancer Institute and Harvard T.H. Chan School of Public Health, Boston, MA 02215

83) Center for Public Health Genomics, University of Virginia, PO Box 800717, Charlottesville, VA 22908-0717, USA
84) Molecular Pathology Unit & Cancer Center, 55 Fruit St Boston MA 02114
85) Department of Biostatistics and Bioinformatics, Moffitt Cancer Center, 12902 Magnolia Drive, Tampa, FL 33612
86) Institute for Stem Cell Biology and Regenerative Medicine, Stanford University, 265 Campus Drive, Stanford, CA 94305
87) Department of Medicine, Brigham and Women's Hospital and Harvard Medical School, 77 Ave Louis Pasteur, Boston, MA 02215
88) Department of Statistics, Medical Sciences Center, 1300 University Ave, Madison, WI 53706 USA
89) Department of Biostatistics and Medical Informatics, 425 Henry Mall 2130 Genetics/Biotechnology Center, Madison, Wisconsin 53706, USA
90) Department of Mathematical Sciences at University of Texas at Dallas, 800 W Campbell Rd, FO 3.608 Richardson, TX 75080
91) Department of Statistics, University of Nebraska-Lincoln, 354C Hardin Hall North Wing, Lincoln, NE 68583-0963 USA
92) The Ohio State University, Department of Biomedical Informatics, 1800 Cannon Drive, 250 Lincoln Tower, Columbus, OH 43210
93) Department of Cell and Regenerative Biology, UW-Madison Blood Research Program, Carbone Cancer Center, University of Wisconsin School of Medicine and Public Health, University of Wisconsin, Madison, Wisconsin 53705, USA
94) Department of Genetics and Genomic Sciences, Icahn School of Medicine at Mount Sinai, 1 Gustave L Levy Place, New York, NY 10029
95) Wellcome Sanger Institute, Cambridge, CB10 1 SA, UK
96) Program in Computational Biology, Memorial Sloan Kettering Cancer Center, 1275 York Ave, New York, NY 10065
97) 610 Charles E. Young Drive S, Terasaki Life Sciences Building, Room 2000E, Los Angeles, CA 90095-1570
98) McKusick-Nathans Institute of Genetic Medicine, Johns Hopkins University, Baltimore, Maryland, USA
99) Department of Biomedical Engineering, Johns Hopkins University, Baltimore, Maryland, USA
100) University of California, Santa Cruz, California 95064, USA

101) Center for Integrative Genomics, University of Lausanne, 1015 Lausanne, Switzerland
102) Centro Nacional de Investigaciones Cardiovasculares (CNIC) and CIBER de Enfermedades Cardiovasculares (CIBERCV), Madrid, Spain
103) Spanish National Cancer Research Centre (CNIO), E-28029 Madrid, Spain
104) Brunel University London, London, UB8 3PH, UK
105) King’s College London, Guy's Hospital, Great Maze Pond, London, SE1 9RT, UK
106) ELIXIR Hub, Wellcome Genome Campus, Hinxton, Cambridge, CB10 1SD, UK
107) Institute of Cancer Research, Chester Betty Labs, South Kensington, London, SW3 6JB, UK
108) Department of Biological Science, 319 Stadium Drive, Florida State University, Tallahassee, FL 32306, USA
109) Department of Biochemistry, Molecular Biology and Biophysics, University of Minnesota Medical School, Minneapolis, MN 55455
110) Center for Vaccines and Immunology University of Georgia, CVI Room 1506, 501 DW Brooks Drive, Athens, GA 30602, USA
111) Center for Molecular Medicine and Department of Biochemistry and Molecular Biology, University of Georgia, Athens, GA 30602, USA
112) Gift of Life Donor Program, 401 North 3rd Street, Philadelphia, PA 19123
113) American Society for Radiation Oncology, 251 18th Street South, 8th Floor, Arlington, VA 22202
114) National Cancer Institute, National Institutes of Health, 9609 Medical Center Drive, Bethesda, MD 20892
115) Leidos Biomedical, Inc., 8560 Progress Drive, Frederick, MD 21701
116) National Disease Research Interchange (NDRI), 8 Penn Center, 15th Floor, 1628 JFK Boulevard, Philadelphia, PA 19103
117) 4407 Puller Dr, Kensington, MD 20895

Contact author: mpsnyder@stanford.edu

## Supplementary Note 1 Useful URLs

**ENCODE Portal**

a) ENCODE Portal: https://www.encodeproject.org

**Using ENCODE Data**

b) ENCODE Data use policy: https://www.encodeproject.org/about/data-use-policy/

c) ENCODE Tutorials and handouts: https://www.encodeproject.org/tutorials/
https://www.encodeproject.org/tutorials/

**Accessing ENCODE data**

d) ENCODE encyclopedia: https://www.encodeproject.org/data/annotations/

e) ENCODE SCREEN: http://screen.encodeproject.org

**f)** ENCODE Registry of candidate regulatory elements:
https://www.encodeproject.org/matrix/?type=Annotation&annotation_type=candidate+regulatory+elements&files.file_type=bed+bed3%2B

**g)** Factorbook of transcription factors assayed by ENCODE: http://factorbook.org

**h)** REST API: https://www.encodeproject.org/help/rest-api/

**i)** All ENCODE data:
https://www.encodeproject.org/matrix/?type=Experiment&award.project=ENCODE

1. all ENCODE mouse data:
https://www.encodeproject.org/matrix/?type=Experiment&award.project=ENCODE&replicates.library.biosample.donor.organism.scientific_name=Mus+musculus
2. all ENCODE human data:
https://www.encodeproject.org/matrix/?type=Experiment&award.project=ENCODE&replicates.library.biosample.donor.organism.scientific_name=Homo+sapiens
3. all modENCODE/modERN data:
https://www.encodeproject.org/matrix/?type=Experiment&award.project=modENCODE&award.project=modERN
    i. all worm data:
    https://www.encodeproject.org/matrix/?type=Experiment&award.project=modENCODE&award.project=modERN&replicates.library.biosample.donor.organism.scientific_name=Caenorhabditis+elegans
    ii. all fly data:
https://www.encodeproject.org/matrix/?type=Experiment&award.project=modENCODE&award.project=modERN
4. ENCODE EN-TEx samples:
https://www.encodeproject.org/matrix/?searchTerm=gtex&type=Experiment&status=released&replicates.library.biosample.biosample_type=tissue&y.limit=
5. ENCODE 3 mouse matrix samples:
https://www.encodeproject.org/matrix/?searchTerm=ENCODE+3&type=Experiment&award.rfa=ENCODE3&replicates.library.biosample.donor.organism.scientific_name=Mus+musculus&biosample_type=tissue&organ_slims=brain&organ_slims=heart&organ_slims=liver&organ_slims=limb&organ_slims=epithelium&organ_slims=lung&organ_slims=stomach&organ_slims=kidney&organ_slims=intestine&organ_slims=embryo

**Examples of how ENCODE data are used**

j) Community and ENCODE consortium publications: https://www.encodeproject.org/publications/

**How ENCODE data are generated and processed**
k) ENCODE Experimental guidelines/protocols and data standards/quality metrics: https://www.encodeproject.org/data-standards/
l) ENCODE data processing pipelines: https://www.encodeproject.org/pipelines/
m) Software tools: https://www.encodeproject.org/software/
n) Ontologies used by ENCODE: https://www.encodeproject.org/help/getting-started/#Ontologies

1. experimental assay types (OBI: http://obi-ontology.org/page/Main_Page)
2. cell lines (EFO: http://www.ebi.ac.uk/efo/)
3. primary cells (CL: http://cellontology.org/)
4. tissues (UBERON: http://uberon.org/)

o) Antibody characterization: https://www.encodeproject.org/search/?type=AntibodyLot
p) ENCODE Help Desk: encode-help@lists.stanford.edu

**ENCODE Participants**
q) ENCODE 3 participants: https://www.genome.gov/27568353/encode-phase-iii-participants-and-projects/
r) ENCODE 4 participants: https://www.genome.gov/26525220/encode-participants-and-projects/

**ENCODE is a member of these organizations**
s) International Human Epigenome Consortium (IHEC): http://ihec-epigenomes.org/welcome/
t) Global Alliance for Genomics and Health (GA4GH): https://www.ga4gh.org

**ENCODE Tissues and Cell Lines: Current and Planned**
u) https://www.encodeproject.org/proposed-biosamples/

**Related Projects**
v) NIH Roadmap Epigenomics Program (REMC): https://commonfund.nih.gov/epigenomics/index
w) International Human Epigenome Consortium (IHEC): http://ihec-epigenomes.org
x) Canadian Epigenetics, Environment and Health Research Consortium (CEEHRC) Network: http://www.epigenomes.ca
y) Blueprint: http://www.blueprint-epigenome.eu
z) 4D Nucleome Program (4DN): https://commonfund.nih.gov/4Dnucleome/index
aa) The Cancer Genome Atlas (TCGA): https://cancergenome.nih.gov
bb) Genotype and Tissue Expression Project (GTEx): https://commonfund.nih.gov/GTEx/index
cc) PsychENCODE: https://www.nimhgenetics.org/available_data/psychencode/
dd) Functional Annotation of the Mammalian Genomes (FANTOM) https://fantom.gsc.riken.jp/
ee) The Human Cell Atlas (HCS) https://www.broadinstitute.org/research-highlights-human-cell-atlas
ff) The Human Biomolecular Atlas Program (HuBMAP) https://commonfund.nih.gov/hubmap
gg) Functional Annotation of Animal Genomes (FAANG): http://www.faang.org
